# A Review of Computational Modeling of Polymer Composites and Nanocomposites

**DOI:** 10.3390/polym18040443

**Published:** 2026-02-10

**Authors:** Zhangke Yang, Zhaoxu Meng

**Affiliations:** Department of Mechanical Engineering, Clemson University, Clemson, SC 29634, USA

**Keywords:** polymer composite, polymer nanocomposite, multiscale modeling, representative volume element, molecular dynamics, coarse-grained molecular dynamics

## Abstract

Polymer composites and nanocomposites have become indispensable in aerospace, automotive, energy, electronics, soft robotics, and biomedical applications due to their high specific stiffness, strength, and manufacturability with highly tailorable multifunctional performance. Their rational design is complicated by strong, multiscale couplings among microstructural heterogeneity, interfacial physics, anisotropic response, and time- and temperature-dependent behavior, spanning molecular to structural length scales. This review provides a comprehensive survey of the principal computational methodologies used to predict and interpret the mechanical behavior of polymer composites and nanocomposites, highlighting the capabilities, specialties, and complementary roles of different modeling tools. This review first summarizes the essential physical characteristics governing polymer composites and nanocomposites. We then examine computational modeling approaches for polymer composites across four length scales: the constituent scale, microscale, mesoscale, and macroscale. For each scale, the primary modeling objectives, characteristic capabilities, and domains of applicability are discussed in the context of the existing literature. Cross-scale relationships and bridging strategies among these scales are also discussed, emphasizing how lower-scale simulations inform higher-scale models. The review then focuses on computational modeling of polymer nanocomposites, with particular attention to atomistic and coarse-grained molecular dynamics methods. Representative atomistic simulations, which capture interfacial structure, reinforcement–matrix interactions, and nanoscale mechanisms, are discussed. This is followed by discussions on coarse-grained approaches that extend the accessible length and time scales. Finally, we discuss how atomistic and coarse-grained models complement each other within integrated multiscale frameworks, enabling predictive links between nanoscale physics and macroscopic mechanical behaviors.

## 1. Introduction

Polymer composites and nanocomposites have emerged as one of the most versatile classes of engineered materials over the past three decades, driven by their unique combination of low density, manufacturability, and tunable mechanical and multifunctional properties. Unlike monolithic metals or ceramics, the integration of polymers with reinforcing phases such as carbon fibers, platelets, or nanoparticles enables dramatic enhancements in stiffness, strength, toughness, electrical conductivity, or thermal performance that far exceed what the polymer matrix alone can offer [1,2]. These improvements have enabled widespread industrial adoption in aerospace structures [3], automotive components [4], wearable electronics [5], energy storage [6], soft robotics [7], and biomedical systems [8]. Despite these advances, polymer composites and nanocomposites inherently exhibit nonhomogeneous, anisotropic, and hierarchical properties, which complicate their design and performance optimization [9,10]. Computational modeling has therefore become a critical tool for characterizing and predicting the complex physical behaviors of polymer composites across various scales.

Historically, research in polymer composites first relied on meso- to large-scale fillers such as carbon fibers, glass fibers, or ceramic particulates. While these systems significantly improved structural performance, their reinforcement efficiency was limited due to the modest surface-to-volume ratio of micron-sized inclusions [9]. The development of nanofillers, such as carbon nanotubes (CNTs), graphene, 2D clays, and inorganic nanoparticles, revolutionized this field. The large interfacial area of nanofillers fundamentally altered the interaction between polymer chains and filler surfaces, enabling dramatic improvements in stiffness, toughness, and multifunctional transport properties at very low volume fractions [11,12]. Crucially, the physics governing these improvements are inherently nanoscale and interfacial: restricted polymer mobility, chain adsorption, nanoscale confinement, and surface functionalization create interphase regions with properties distinctly different from both the bulk polymer and the filler [13,14]. A purely macroscale continuum mechanics framework is insufficient to capture these phenomena; rigorous understanding and predictive modeling require explicit incorporation of nanoscale and mesoscale descriptions that resolve underlying molecular interactions, interfacial heterogeneity, and microstructural evolution.

The hierarchical nature of polymer composites makes computational modeling uniquely challenging. The mechanisms that govern performance span multiple orders of magnitude in both length and time: molecular chain entanglement and adsorption (Å to nm), nanoparticle dispersion and agglomeration (nm to µm), microstructural organization in representative volume elements (µm to mm), and structural load-bearing responses at the device level (mm to m). Multiscale modeling frameworks are, therefore, necessary to bridge information across regimes. Atomistic simulations such as molecular dynamics (MD) and density functional theory (DFT) are indispensable for studying interfacial energetics, chain pull-out, adsorption, and cross-linking chemistry [15,16]. Coarse-grained (CG) models and dissipative particle dynamics (DPD) enable the exploration of microscale to mesoscale morphology evolution, nanoparticle dispersion, and self-assembly beyond the temporal limits of MD [17,18,19,20,21,22,23]. At larger scales, micromechanics [24], discrete mesoscale modeling (DMM) [25], and finite element modeling (FEM) [26,27] are employed to predict effective elastic constants, localization phenomena, damage initiation, and nonlinear constitutive responses.

Each of these modeling approaches provides valuable yet inherently incomplete insight into the behavior of polymer composites and nanocomposites, reflecting the fundamentally different spatial and temporal scales at which they operate. Quantum and atomistic-level approaches accurately resolve interfacial energetics, local chain conformations, and chemical specificity, but their prohibitive computational cost precludes simulations of large systems or long-time structural evolution. CG and mesoscale methods extend accessible length and time scales, enabling representation of nanoparticle dispersion, aggregation, and phase morphology; however, this scalability is achieved at the expense of atomic detail and direct chemical fidelity. Continuum micromechanics and finite element models, in turn, provide efficient predictions of effective properties and macroscopic structural responses. Yet, they depend on prescribed constitutive relations, obscure critical small-scale mechanisms, and are not well-suited for capturing discontinuous behaviors such as damage initiation and fracture. The central challenge, therefore, lies in developing rigorous, self-consistent strategies that couple these modeling levels, allowing mechanistic information to flow both upward and downward across scales. Overcoming this challenge remains a key bottleneck to achieving truly predictive, design-oriented modeling frameworks for polymer composites and nanocomposites.

More recently, machine learning (ML) and AI-accelerated surrogate modeling have begun reshaping computational research on polymer nanocomposites. Data-driven convolutional neural networks (CNNs), physics-informed neural networks (PINNs), and generative models have demonstrated the ability to predict mechanical, thermal, and multiphysics responses orders of magnitude faster than traditional simulation pipelines while retaining fidelity to complex microstructure–property relations [28,29]. Such approaches have also expedited inverse material design, where microstructure and filler distributions are optimized automatically to achieve target properties [30]. When coupled with physically grounded simulation frameworks, AI provides an opportunity to unify the multiscale hierarchy of polymer composites and nanocomposites and accelerate their computational discovery and optimal design.

In this review, we survey the current landscape of computational modeling approaches for polymer composites and nanocomposites. We begin by outlining the fundamental physical characteristics of polymer matrices and reinforcements in both conventional composites and nanocomposite systems. We then examine modeling strategies spanning atomistic, mesoscale, and continuum frameworks, emphasizing their respective capabilities and limitations. Finally, we highlight emerging AI-guided and physics-informed ML approaches that enable efficient predictive simulation, optimization, and inverse design. By integrating these perspectives, this review seeks to provide a coherent roadmap for advancing simulation methodologies and multiscale strategies that can faithfully capture structure–composition–property relationships in advanced polymer composite and nanocomposite systems. While several reviews have addressed specific aspects of computational modeling of polymer composites and nanocomposites, the present work aims to provide a more integrated and mechanistically grounded perspective. In contrast to prior reviews that primarily focus on individual modeling scales or specific techniques, this review systematically organizes atomistic, mesoscale, and continuum modeling approaches within a unified multiscale framework, explicitly emphasizing their interconnections and information transfer across length scales. Particular attention is given to elucidating how nanoscale interactions, interfacial phenomena, and microstructural features influence macroscopic mechanical and functional properties. Furthermore, this review offers an integrated discussion of conventional physics-based modeling approaches alongside emerging artificial intelligence and machine-learning-based surrogate models, highlighting their complementary roles, limitations, and opportunities for cross-scale integration. Through this systematic synthesis, the present review aims to advance mechanistic understanding and provide clearer guidance for the rational design and predictive modeling of polymer composites and nanocomposites.

## 2. Fundamentals of Polymer Composites and Nanocomposites

In this review, polymer composites and polymer nanocomposites are distinguished based on the characteristic length scales of their reinforcements and the resulting dominant physical mechanisms. Polymer composites typically consist of a polymer matrix reinforced with fibers or particulates whose dimensions are on the order of micrometers or larger, such that their mechanical behavior can often be described using continuum-based micromechanics, representative volume elements, and homogenization approaches. In contrast, polymer nanocomposites incorporate reinforcements with at least one characteristic dimension in the nanometer range, leading to a significantly increased interfacial area between the reinforcement and the matrix. As a result, nanoscale effects, such as interphase formation, confinement-induced changes in polymer chain dynamics, and dispersion or agglomeration of nanofillers, play a critical role in governing mechanical response. These fundamental differences in length scale and underlying physics necessitate distinct modeling assumptions and multiscale strategies, which are discussed throughout this review.

Polymer composites and nanocomposites derive their unique mechanical, thermal, and multifunctional capabilities from the interplay between the polymer matrix, reinforcing phases, and the interphase that mediates their interactions. Their performance reflects a hierarchical organization of molecular, nanoscale, microscale, and macroscale features. This section discusses the essential physical characteristics governing polymer composites and nanocomposites, including polymer matrix behavior, reinforcement characteristics, interphase region, and multiscale architectures. These fundamentals establish the physical basis for computational models described in later sections.

### 2.1. Polymer Matrix

The polymer matrix constitutes the continuous phase that embeds and supports the reinforcements. Owing to its complex molecular architecture, characterized by chain entanglements, segmental relaxations, free-volume fluctuations, and often partial crystallinity, the polymer exhibits inherently nonlinear and time-dependent mechanical behavior.

#### 2.1.1. Molecular Motion and Relaxation

Polymer mechanics arises from molecular motions at multiple length scales. At the atomistic scale, the local stiffness is influenced by the ultrafast motions, such as bond vibrations and torsional rotations [31,32]. At the nanoscale, the segmental α-relaxations govern the glass transition and viscoelastic response [33,34]. At the microscale, the chain reptation and disentanglement affect yield, flow, and long-time relaxation [35,36]. Moreover, temperature, strain rate, and degree of cross-linking strongly impact these processes [37,38].

#### 2.1.2. Influence of Confinement

Polymer chains in the vicinity of surfaces or fillers exhibit restricted mobility, modified relaxation spectra, local densification or structural ordering, and corresponding shifts in glass-transition temperature [39,40,41,42]. These confinement effects fundamentally shape interphase behavior and directly influence macroscopic properties. Reinforcements and nanofillers also impose confinement on the surrounding polymer matrix, typically increasing the overall stiffness [19,23,43], in some specially designed cases, enabling materials to surpass the conventional stiffness-damping trade-off observed in traditional systems [23].

#### 2.1.3. Mechanical Properties

The polymer matrix plays an important role in governing the macroscopic mechanical performance of composites and nanocomposites [44]. Its intrinsic viscoelasticity provides significant energy dissipation capability through polymer chain sliding, which is essential for damping and impact resistance [45,46]. The polymer matrix also retards crack propagation and serves as the primary source of toughness, primarily through mechanisms such as crack blunting, shear yielding, localized plastic deformation, and effective stress redistribution [47,48,49]. In addition, the matrix controls interfacial interaction with reinforcements, whether fibers, platelets, tubes, or nanoparticles, which determines the efficiency of load transfer, prevents premature debonding, and influences stiffness, damping, and strength of the composite or nanocomposites [13,50].

### 2.2. Reinforcements

Reinforcements play a decisive role in determining the stiffness, strength, toughness, and functional behavior of polymer composites and nanocomposites [51,52,53]. Their contributions arise not only from their intrinsic mechanical properties, such as modulus, strength, and resilience, but also from their interactions with the surrounding matrix.

#### 2.2.1. Reinforcements of Composites

Conventional reinforcements, such as carbon fibers [54], glass fibers [55], ceramic whiskers [56], and particulates [57], have long served as the backbone of high-performance polymer composites. These reinforcements typically possess elastic moduli and strengths that are much higher than those of the surrounding polymer matrix, enabling significant enhancement of the composite’s overall stiffness, strength, and load-bearing capability. Mechanically, reinforcements improve the performance of composites primarily through efficient load transfer [58], crack bridging [59], fiber pull-out toughening [60], and crack deflection and branching [49].

#### 2.2.2. Reinforcements of Nanocomposites

Nanocomposite reinforcements, such as carbon nanotubes [61], graphene [19,62], and other two-dimensional nanosheets [63], nanoclays [64], nanocellulose [65], and nanoparticles [23,66], exhibit strong interactions with the polymer matrix and can therefore impart exceptional mechanical performance. Their effectiveness typically arises from exceptionally high surface-to-volume ratios, large aspect ratios, nanoscale confinement effects, and, in some cases, intrinsic multifunctional properties. As a result, these reinforcements enable significant property enhancements even at relatively low volume fractions through critical interfacial interactions and the formation of heterogeneous or percolated reinforcing networks.

#### 2.2.3. Influence of Dispersion and Orientation

The reinforcing effect of fillers in polymer composites and nanocomposites is strongly governed by their state of dispersion and orientation within the polymer matrix. Generally, uniform dispersion and effective avoidance of agglomeration are critical to fully exploiting the intrinsic properties of reinforcements. However, in certain cases, a deliberately nonuniform distribution of reinforcements can be used to induce heterogeneous or anisotropic mechanical properties in composites and nanocomposites, particularly in the latter. Such spatially tailored architectures can be highly advantageous for designing adhesive and interfacial materials that bridge mechanically dissimilar constituents, as they enable improved stress transfer and mitigation of interfacial stress concentrations [25].

Beyond dispersion, the orientation of reinforcements also plays an important role in determining macroscopic composite behavior. Randomly oriented fillers generally lead to isotropic mechanical and functional responses, whereas aligned or preferentially oriented high-aspect-ratio reinforcements, such as fibers, nanotubes, or two-dimensional nanosheets, can induce pronounced anisotropy in stiffness, strength, fracture resistance, and other functional properties [12,25,67,68]. Such anisotropic responses are often highly desirable in engineered composites [69], tissue engineering constructs [70], and biomimetic scaffolds [27], as they enable tailored load-bearing capacity, directional properties, and controllable failure behavior.

### 2.3. Interphase Region

The interphase region is the zone of altered polymer structure and properties that forms in the vicinity of reinforcements as a result of interfacial interactions, geometric confinement, and processing history [13,71]. Unlike the idealized sharp interface assumed in classical composite theory, the interphase in polymer composites and nanocomposites typically extends over a finite thickness and exhibits material properties that differ from both the bulk polymer matrix and the reinforcement [72]. This interphase plays a critical role in governing load transfer, stress distribution, viscoelastic response, damage initiation, and failure evolution, and is therefore a key determinant of composite performance.

Interphase region formation originates from physical and chemical interactions between the polymer and the reinforcement surface. These interactions include van der Waals forces [61,73], electrostatic interactions [61], hydrogen bonding [61,73], and in some systems, covalent bonding or grafting [74]. Attractive interactions promote polymer adsorption onto filler surfaces, leading to local densification, chain immobilization, and modified segmental dynamics [23]. In contrast, weak or repulsive polymer–reinforcement interactions can lead to polymer depletion near the reinforcement surface [75], reduced interfacial adhesion [76], and an increased tendency for interfacial debonding under mechanical loading [77].

Geometric confinement imposed by reinforcements also strongly influences interphase structure. Polymer chains near reinforcement surfaces experience restricted configurational freedom [13], altered entanglement density [78], and modified relaxation spectra [79]. These confinement effects can lead to either stiffened or softened interphase regions depending on the balance between chain immobilization, free-volume changes, and local packing frustration [13,80,81]. Processing conditions in composites and nanocomposites also play a decisive role in interphase development. Thermal history, flow, and shear during mixing or forming, and curing reactions in thermosets can all modify polymer adsorption and mobility near reinforcement surfaces, regulate interfacial or transcrystalline crystallization, and ultimately govern the formation and quality of interfacial bonding [9,13,82]. Mechanically, the interphase mediates stress transfer between the polymer matrix and the reinforcement. A stiff, well-bonded interphase improves interfacial load transfer and typically increases composite stiffness and strength. In contrast, a more compliant or weakly bonded interphase can activate interfacial sliding and debonding-related dissipation mechanisms that enhance toughness and damping, but it may also reduce stiffness and promote premature interfacial failure under load [13,83].

Recent studies increasingly treat the reinforcement–matrix interphase as a physically distinct region whose chemistry, mobility, and effective properties can dominate macroscopic response, especially in nanocomposites where the interfacial area is large. On the modeling side, current work has moved beyond idealized “perfect bonding” assumptions toward explicit interphase descriptions, enabling more realistic predictions of stiffness/strength and coupled functional behavior when dispersion and clustering are present [84,85,86]. In parallel, characterization advances are improving the quantification of interphase thickness and local properties, including practical approaches for estimating interphase presence/thickness from accessible measurements and emerging frameworks for extracting interphase mechanical properties from high-resolution mechanical probing/indentation-type measurements [87,88]. Together, these developments support a more mechanism-based interphase-centric view in which experimentally informed interphase descriptors are integrated into micromechanical or multiscale models to capture reinforcement–matrix load transfer and damage sensitivity in a way that is not attainable with interfaceless homogenization alone [86,89].

Overall, the interphase plays a central role in linking nanoscale interactions to macroscopic composite behavior. Consequently, understanding and controlling the interphase is essential for predictive modeling and the rational design of polymer composites and nanocomposites.

### 2.4. Multiscale Architectures

Polymer composites and nanocomposites possess inherently multiscale architectures, ranging from nanoscale features at the polymer–reinforcement interface to microscale reinforcement networks and macroscopic structural organization. These multiscale architectures fundamentally govern composite behavior, since mechanical, thermal, and functional responses are not localized at a single scale but instead emerge from their coupled interactions across multiple length scales [90].

At the nanoscale, composite architecture is governed by reinforcement dimensions and morphology, surface chemistry, and the resulting dispersion state within the polymer matrix. The spatial distribution of reinforcements, including their spacing, aggregation, and connectivity, controls interphase formation and confinement effects, promotes or suppresses percolated filler networks, and shapes local stress/strain concentration patterns that influence macroscopic mechanical and functional response [11,91,92]. Even modest changes in nanoscale reinforcement morphology, including aggregation, surface corrugation/roughness, or sheet wrinkling, can significantly alter interphase chain dynamics and interfacial load transfer, thereby reshaping the dominant energy-dissipation pathways and the resulting mechanical and viscoelastic responses [23,93]. As a result, nanoscale architectural features strongly affect effective material properties despite representing only a small fraction of the composite volume.

At the microscale, reinforcements form networks, clusters, or aligned domains that govern stress redistribution, damage evolution, and anisotropy [94]. The reinforcement spacing, orientation distribution, and network connectivity together establish the characteristic length scales for load transfer, govern preferred crack-deflection trajectories, and regulate the dominant deformation and damage mechanisms [95,96,97].

At the macroscale, composite architecture encompasses laminate stacking sequences, layered and functionally graded designs, architected cellular or lattice structures, and spatially programmed reinforcement content and/or orientation enabled by processing routes and additive manufacturing [98,99,100]. These macroscopic architectural choices largely determine global stiffness, strength, stability (e.g., buckling resistance), damage tolerance, and dominant failure modes, while also constraining and amplifying how micro- and nanoscale mechanisms (e.g., matrix yielding, interfacial debonding, and delamination) manifest at the structural level [101,102,103].

Most importantly, these length scales are not independent. Nanoscale interphase properties influence microscale load transfer, deformation, and damage initiation, which in turn govern macroscopic strength, toughness, and reliability. Conversely, macroscopic architecture and processing impose mechanical, thermal, and chemical boundary conditions at smaller scales, shaping microstructural evolution and interfacial behavior. Composite performance therefore emerges from a continuous cascade of coupled structure–composition–property relationships across length scales. Polymer composites and nanocomposites are therefore most appropriately analyzed within multiscale frameworks, where architecture acts as the central link connecting chemistry, morphology, mechanics, and function.

## 3. Computational Modeling of Polymer Composites

Polymer composites have become an indispensable class of structural and functional materials due to their high specific stiffness and strength, design flexibility, and broad applicability across aerospace, automotive, civil, and biomedical engineering [8,104,105]. The inherently heterogeneous mechanical properties of polymer composites, arising from the coexistence of polymer matrices and reinforcing phases, pose significant challenges for experimental characterization alone, particularly in elucidating the underlying small-scale deformation and failure mechanisms. Owing to its efficiency, cost-effectiveness, and ability to capture details, computational modeling has emerged as a critical tool for understanding structure–property–performance relationships in polymer composites and for guiding their rational design. This section focuses on the computational modeling of polymer composites, with emphasis on continuum-based frameworks that have been extensively developed and applied over the past several decades and proven to be both efficient and robust.

### 3.1. Overview of Computational Modeling of Polymer Composites

Continuum-based computational modeling has long served as a cornerstone for analyzing the mechanical behavior of polymer composites at engineering length scales. This approach enables efficient simulation of macroscopic responses while accounting for microscale heterogeneity either explicitly or implicitly, depending on the modeling strategies. Among continuum methods, the finite element method (FEM) [106,107,108] is by far the most widely used due to its capability in handling complex geometries, boundary conditions, and nonlinear material behavior. FEM-based models have been extensively used to study elastic and inelastic deformation [109,110], fracture [110], viscoelasticity [111], and thermomechanical coupling [112] in polymer composites. By incorporating appropriate constitutive laws and failure criteria, continuum models can capture a wide range of mechanical behaviors of composites under diverse loading conditions. Continuum-based models are particularly advantageous in terms of computational efficiency and scalability, making them well-suited for parametric studies, structural-scale simulations, and design optimization. Continuum models typically involve far fewer degrees of freedom, and therefore substantially lower computational cost, than atomistic or fully discrete approaches. Owing to their maturity, robustness, and reliability, continuum-based methods have been widely used in industrial settings, including component-level simulations of aerospace and automotive composite structures [109,113,114]. Despite these strengths, continuum approaches inherently rely on assumptions of scale separation [115] and the existence of an effective or homogenized material response [116]. As a result, their predictive capability depends critically on the accuracy of material constitutive models. Consequently, continuum-based modeling is often complemented by experiments or lower-scale simulations to inform and calibrate the material constitutive models. Overall, for predicting the macroscopic mechanical response of polymer composites, continuum-based computational modeling, particularly FEM, remains one of the most robust and efficient tools.

### 3.2. Length Scales and Modeling Methods

**Figure 1:**
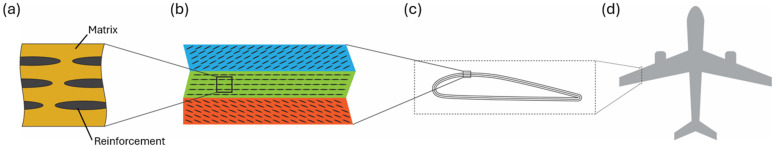
Schematics showing the characteristic length scales of polymer composites: (**a**) constituent scale, (**b**) microscale, (**c**) mesoscale, and (**d**) macroscale.

Polymer composites consist of a polymer matrix and reinforcing phases, whose spatial arrangement typically occurs at length scales much smaller than that of the composite component yet strongly influences the overall mechanical response. As a result, polymer composites exhibit an inherently multiscale nature, encompassing the constituent scale, microscale, mesoscale, and macroscale, as illustrated in Figure 1. Computational modeling at each scale captures the dominant mechanical mechanisms operative at that length scale, and the corresponding models may be analyzed independently or in coupled manners, depending on practical needs and specific research objectives.

At the constituent scale, which is the smallest relevant length scale, research focuses on the fundamental components of the composite, namely the polymer matrix and the reinforcing fibers or particles. The mechanical behavior of polymer composites at this scale is governed by intrinsic material properties, including matrix elasticity or viscoelasticity, fiber stiffness and strength, and interfacial phenomena such as adhesion, debonding, and sliding. Computational modeling at the constituent scale is primarily aimed at elucidating the mechanisms governing local stress transfer, damage initiation, and failure processes that arise from the matrix–reinforcement interactions. Although continuum descriptions may still be employed at this scale, constitutive models are typically enriched to capture nonlinear material behavior and damage evolution through phenomenological formulations, such as interfacial traction–separation laws. Information obtained at the constituent scale can serve as a critical input for higher-scale models and can also provide guidance for the design of improved matrix-reinforcement interactions.

Unlike the constituent scale, the microscale focuses on a larger representative domain containing a number of reinforcements whose spatial distribution and orientation can be described statistically. This scale explicitly resolves the composite microstructure while remaining sufficiently small relative to the macroscale. Computational modeling at the microscale explicitly represents key microstructural features, including reinforcement geometry, spatial distribution, orientation, and volume fraction, which govern the effective local behavior of the composite. Within a continuum mechanics framework, microscale computational models are often used to obtain homogenized material properties, such as effective elastic, inelastic, or damage-related properties, that can be passed to higher scales. Microscale computational modeling provides an effective bridge between constituent-level mechanisms and macroscopic composite behavior, while remaining computationally efficient compared with fully discrete or atomistic approaches.

At the mesoscale, computational modeling focuses on intermediate structures such as plies, laminates, woven fabrics, or architected composite layouts. At this scale, the composite is not assumed to be fully homogenized but is instead represented as an assembly of substructures or layers with distinct, typically anisotropic, effective properties. It is very important for mesoscale computational models to capture anisotropy, interply interactions, and damage modes such as delamination or ply cracking. The layered structural features at the mesoscales are commonly captured using finite element formulations with layered shell or solid elements, while the associated material properties are usually supplied by microscale homogenization procedures or experimental characterization.

The macroscale corresponds to full structural components or engineering systems, where the composite is typically treated as an effective homogeneous or layered continuum. At this scale, the primary goal of computational modeling is typically to predict global mechanical responses, such as deformation and vibration, under realistic loading and boundary conditions. Macroscale computational models enable component-level simulations essential for engineering design, particularly in aerospace, automotive, and civil applications, where polymer composites are widely used owing to their superior properties. The accuracy of macroscale predictions depends critically on the fidelity of the underlying material constitutive models, which are commonly derived from lower-scale simulations or experimental measurements.

### 3.3. Modeling of Polymer Composites at the Constituent Scale

At the constituent scale, typically ranging from $10^{-9}$ to $10^{-5}$ m, computational modeling focuses on the mechanical behavior of the individual phases, namely polymer matrix and reinforcements, as well as their interactions. Accurate representation of the polymer matrix is the key in constituent-scale modeling, as polymers often exhibit nonlinear, time-dependent mechanical responses that establish the baseline performance of the composite. Among all those constitutive models, linear elastic formulations are the simplest and are appropriate for small deformations and for cases in which the response remains in elastic regimes [117]. However, most polymers exhibit nonlinear elastic behavior and viscosity; therefore, nonlinear viscoelastic constitutive models are commonly employed to describe their mechanical response. Tee and Dealy [118] conducted one of the early systematic studies of nonlinear viscoelasticity of polymer melts under large-amplitude oscillatory shear (LAOS). They examined three common thermoplastic melts, HDPE, LDPE, and polystyrene (PS), to compare nonlinear response characteristics across materials. Tervoort et al. [119] developed a three-dimensional, multi-mode constitutive model to describe the finite nonlinear viscoelastic behavior of polymer glasses up to yield. The model assumes that nonlinearity arises primarily from a stress-dependent acceleration of the linear relaxation spectrum, implemented through a time–stress superposition concept. A multi-mode formulation is used to represent the relaxation spectrum realistically and avoid the abrupt transitions associated with single-mode models. The framework was validated using creep and constant strain-rate experiments on polycarbonate, showing good agreement up to yield and supporting the applicability of stress-controlled relaxation shifting in glassy polymers. Drozdov and Kalamkarov [120] proposed a small-strain constitutive framework for noncrosslinked polymers that extends adaptive-network ideas to describe nonlinear viscoelastic response within a continuum formulation. While nonlinear viscoelastic models capture time- and rate-dependent but recoverable deformation, many polymers also exhibit irreversible flow and permanent deformation under sufficiently high stresses or long loading durations, motivating the use of nonlinear viscoplastic models to describe such inelastic behavior. Zaïri et al. [121] developed a constitutive modeling framework to capture the elasto-viscoplastic response coupled with damage-induced volumetric deformation in glassy polymers, motivated by the observation that the common assumption of isochoric inelastic flow can be violated in rubber-toughened systems due to mechanisms such as crazing, interfacial debonding, and rubber-particle cavitation/void growth.

Beyond the polymer matrix, the mechanical behavior of the reinforcement phase must also be modeled to capture the composite response accurately. Reinforcements in polymer composites are typically much stiffer than the polymer matrix and therefore tend to undergo primarily elastic deformation under typical loading conditions. Accordingly, elastic constitutive models are commonly employed to describe the mechanical response of the reinforcements. Calzada et al. [122] developed a microstructure-resolved finite element model for orthogonal machining of carbon fiber–reinforced polymers, in which carbon fibers were explicitly represented as elastic, anisotropic reinforcements embedded in a polymer matrix. Chawla et al. [123] modeled reinforcement particles by reconstructing their true 3D morphology and distribution from serial sectioning, meshing the resulting angular particle geometries with tetrahedral elements, and treating SiC as linear elastic, demonstrating that such realistic particle representations predict stronger load transfer and more localized matrix plasticity than idealized spherical/ellipsoidal particle models.

At the constituent scale, the matrix–reinforcement interface represents a critical region that governs load transfer and frequently controls damage initiation and failure in polymer composites. Several strategies are commonly used to model this region. The simplest assumes a perfect interface, i.e., no relative displacement occurs across the interface at any time. This assumption is appropriate when the interface is very thin and stronger than both the matrix and the reinforcement. When interfacial strength is finite, or debonding is expected to influence mechanical behavior, more detailed interface representations are required. One widely adopted approach is the use of cohesive zone models (CZMs), which explicitly introduce traction–separation laws to represent progressive debonding and failure at the interface by relating stresses across the interface to relative separations; such models have been extensively employed in the fracture and delamination analysis of composite systems and enable simulation of interfacial crack initiation and growth within a continuum mechanics framework [124]. Alternatively, interphase models treat the interface as a finite-thickness region with graded material properties that reflect altered polymer chemistry or morphology near the reinforcement surface due to processing and adhesion effects. These interphase representations capture spatial variations in mechanical properties around fibers and can significantly influence local stress fields and effective composite response, particularly when adhesion or interfacial chemistry differs from that of the bulk matrix [125].

### 3.4. Microscale Modeling of Polymer Composites

Microscale modeling occupies an intermediate position between the constituent scale and the mesoscale, explicitly resolving a representative portion of the composite microstructure that contains a number of reinforcements embedded within the surrounding polymer matrix. It covers length scales ranging from $10^{-7}$ m to $10^{-4}$ m, depending on the specific application. Unlike constituent-scale models, which focus on the behavior of individual phases, microscale models capture the geometric arrangement, spatial distribution, and orientation of reinforcements in a statistical sense, while remaining sufficiently small relative to the macroscale to allow continuum simulations. By explicitly accounting for microstructural characteristics, this level of modeling enables the derivation of effective composite material properties and provides essential input to hierarchical homogenization and multiscale simulations.

**Figure 2:**
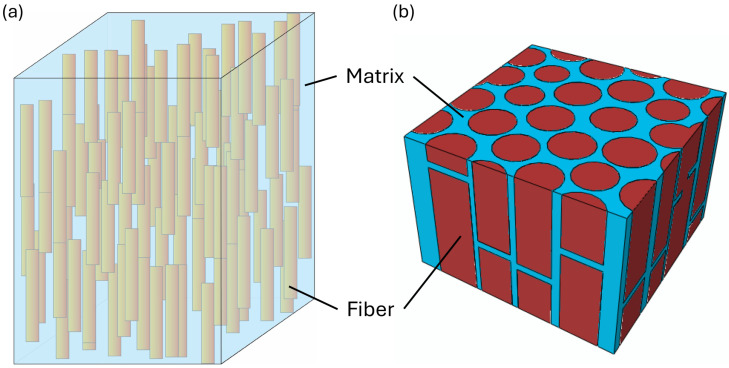
(**a**) Schematic illustration of a polymer composite reinforced with short cylindrical fibers. (**b**) RVE of the polymer composite used for finite-element analysis.

A key concept in microscale modeling of polymer composites is the representative volume element (RVE), defined as the smallest material volume that statistically captures the microstructural heterogeneities of the composite, while its averaged response represents the macroscopic behavior of the material. Typical RVEs for fiber-reinforced polymer composites may consist of an array of fibers embedded in the polymer matrix, as shown in Figure 2a,b, with either periodic or statistically equivalent distributions.

Balasubramani et al. [126] revisited the practical question of how to choose the size and shape of a 3D RVE for unidirectional fiber reinforced polymer composites when the goal is not only homogenized stiffness, but also local-field statistics relevant to strength/damage onset. Using Monte Carlo simulations of random microstructural fiber arrangements, they studied the statistical convergence of both microstructural geometric descriptors and predicted properties, and explicitly compared random 3D RVE predictions against commonly used periodic unit-cell idealizations. Wang et al. [127] present an RVE-based multiscale framework to predict thermo-oxidative aging effects in particle-filled polymer composites by explicitly embedding aging-dependent matrix and interface properties into a microscale model. The authors construct an RVE using a random particle-packing algorithm and apply uniform displacement boundary conditions to simulate tensile loading. Trias et al. [128] addressed RVE selection for carbon fiber–reinforced polymers by introducing the concept of a statistical representative volume element (SRVE), motivated by the limitation of many classical RVEs that assume periodic fiber distributions. They argued that, to reproduce microscale damage mechanisms driven by randomness, an SRVE should satisfy both mechanical convergence criteria and point-pattern statistics that reflect the underlying random fiber arrangement.

While microscale modeling yields valuable insight into microscale mechanisms, it is usually embedded within broader multiscale or hierarchical modeling frameworks, in which information from the microscale is passed upward to mesoscale and macroscale models to enable prediction of material behavior at larger length scales [129]. As computational resources and modeling methodologies continue to evolve, research increasingly explores advanced microscale representations, such as image-based geometric models [130], physics-informed surrogate models [131], and ML-assisted homogenization [132], to further enhance predictive accuracy and reduce computational cost.

### 3.5. Mesoscale Modeling of Polymer Composites

Mesoscale modeling serves as a bridge between microscale representations of the reinforcement-matrix system and macroscopic structural behavior by capturing the characteristic architecture and structural heterogeneity of polymer composites. At this scale, typically ranging from $10^{-4}$ m to $10^{-1}$ m, the composite is not considered fully homogenized but is instead represented as an assembly of plies or substructures with distinct orientations, thicknesses, and anisotropic effective properties. By explicitly representing laminate stacking sequences, interply interactions, and in-plane and out-of-plane responses, mesoscale models provide an efficient framework for the practical design and analysis of composite structures in which layered substructures govern the overall behavior.

Finite element methods are the primary computational tools for mesoscale modeling of polymer composites. Based on the structural features at this scale, shell or solid elements are commonly employed to represent individual plies and their interactions. For thin laminated composite layers with similar reinforcement distributions, shell elements are commonly used to efficiently capture in-plane and binding behavior, whereas solid elements are preferred for thick laminates or when detailed through-thickness stress distributions need to be resolved. Mesoscale finite element models employ anisotropic constitutive models for each ply to reflect the spatial distribution of reinforcements, with the corresponding material parameters typically obtained through microscale homogenization or experimental characterization. Computational modeling at this scale is particularly effective for capturing both ply-level mechanical behavior and the influence of stacking sequence on structural response.

Thorsson et al. [133] introduced a shell-based finite element framework to predict the low-velocity impact response of fiber-reinforced polymer (FRP) laminates, with the explicit goal of capturing the dominant intra-laminar and inter-laminar failure mechanisms observed experimentally. The laminate plies are represented with shell elements, while intra-laminar matrix nonlinearity and damage are modeled using Enhanced Schapery Theory to capture matrix microcracking and progressive degradation, together with macroscopic ply failure modes. Inter-laminar damage is treated separately via discrete cohesive zone modeling, enabling initiation and propagation of delamination under impact loading.

Xu et al. [134] developed a continuous-step finite element framework to simulate both low-velocity on-edge impact and the subsequent compression after edge impact response of carbon fiber reinforced plastic quasi-isotropic laminates. In their modeling strategy, the laminate plies are represented using continuum shell elements, and intralaminar damage is governed by the Hashin failure criteria to capture ply-level fiber and matrix failure modes. Interlaminar damage is modeled using zero-thickness cohesive elements, enabling initiation and propagation of delamination under impact-induced stress states and subsequent compressive loading.

Robbins and Reddy [135] developed a layerwise, two-dimensional displacement-based finite element model for thick laminated composite plates based on Reddy’s layerwise laminate theory. The laminate thickness is discretized in a piecewise-continuous manner for transverse strain distributions, allowing the formulation to resolve interlaminar stresses and other localized through-thickness effects with accuracy comparable to conventional 3D solid-element finite element models. A key modeling advantage is that, although the total degrees of freedom can be comparable to a 3D discretization, the proposed approach retains a 2D-type data structure, which simplifies input/meshing and can reduce computational overhead. The authors demonstrated the method on representative laminate problems in bending and extension, highlighting its ability to compute interlaminar stresses accurately.

Krueger and O’Brien [136] developed a global–local shell/3D finite element modeling technique for delaminated composite laminates, aiming to retain the accuracy of full 3D solid models near the delamination front while leveraging the computational efficiency of shell/plate models for the overall structure. In this framework, the global laminate is modeled with shell elements, and a localized 3D solid subregion is introduced only in the immediate vicinity of the delamination front, where the stress state is strongly three-dimensional and fracture parameters must be evaluated accurately. A central modeling feature is the evaluation of delamination driving forces using the Virtual Crack Closure Technique (VCCT), enabling computation of mixed-mode strain energy release rate (SERR) distributions. The shell/3D formulation is shown to capture key 3D effects such as anticlastic bending, which can lead to a maximum SERR at the specimen center across the width.

CZMs are among the most widely used fracture frameworks for polymer composites because they provide a convenient traction–separation representation of the fracture process zone and enable progressive damage simulation within standard finite element implementations. In polymer matrix composites, CZMs are routinely applied to capture delamination and interlaminar cracking by prescribing mode-dependent traction–separation laws calibrated from fracture tests, allowing simulation of initiation and growth under quasi-static and fatigue loading [137]. Beyond delamination, cohesive interfaces are also commonly used to represent fiber–matrix interfacial debonding and pull-out at the microscale, which is critical for predicting load transfer and toughness mechanisms in fiber-reinforced systems [138]. More broadly, the formulation and selection of traction–separation relations (including their strengths, fracture energies, and mixed-mode coupling laws) have been extensively reviewed, providing guidance on model forms, calibration, and limitations for composite fracture applications [139].

Phase-field fracture models are commonly applied to modeling polymer composites, particularly at the laminate and mesoscale, where damage mechanisms such as matrix cracking, delamination, and crack interaction govern the overall failure response. By regularizing sharp cracks through a continuous damage variable, phase-field approaches enable the simulation of crack initiation, propagation, branching, and coalescence without requiring predefined crack paths, which is a significant advantage over interface-based methods in complex loading scenarios [140,141,142]. In composite laminates, these models have been successfully extended to account for material anisotropy, directional fracture resistance, and mixed-mode fracture behavior, allowing realistic representation of fiber orientation effects and ply-level damage evolution. Phase-field formulations also integrate naturally with continuum finite element frameworks and can be coupled with thermo-mechanical or viscoelastic constitutive models, making them well-suited for predicting progressive damage and failure in polymer composite structures under multi-axial and non-uniform loading conditions.

### 3.6. Macroscale Modeling of Polymer Composites

Macroscale modeling of polymer composites focuses on the structural behavior and performance of composite components and assemblies at scales relevant to engineering applications, typically spanning from $10^{-2}$ to $10^{2}$ m. At this scale, detailed micro- and mesoscale features are not resolved explicitly but are instead embedded into effective constitutive descriptions, enabling computationally efficient simulation of large-scale structures such as aircraft wings, automotive panels, and civil infrastructure elements. Macroscale models are employed when interest centers on global stiffness, stability, dynamic response, and structural integrity, rather than on resolving local microstructural fields.

A critical step in macroscale composite modeling is the definition of effective material properties that approximate the behavior of the underlying heterogeneous composite material. These properties are generally inferred from lower-scale analyses and encoded into continuum constitutive laws. For linear elastic composite materials, orthotropic or anisotropic stiffness tensors [143] are commonly used to represent the directional dependence of stiffness induced by the reinforcement architecture. For nonlinear structural responses, such as plasticity, progressive damage, and ultimate failure, continuum mechanics frameworks are commonly extended through internal state variables and associated evolution laws, which introduce additional material parameters to represent strain hardening, stiffness degradation, and the accumulation of damage at the structural scale [144,145].

Macroscale constitutive models of polymer composites may incorporate thermo-mechanical coupling, viscoelasticity, and rate dependence to account for time- and temperature-sensitive polymer behavior commonly observed in engineering applications. Xin et al. [146] proposed a sequential multiscale thermo–mechanically coupled damage modeling framework for continuous carbon-fiber–reinforced thermoplastic composites. Their approach integrates constituent-level calibration, thermo–mechanical constitutive modeling, and homogenization-based upscaling to predict the coupled mechanical response and damage evolution across scales. At the microscale, the thermo–mechanical behaviors of the matrix, carbon fiber bundles, and the fiber–matrix interface were characterized experimentally over a wide range of temperatures and strain rates, and corresponding elastoplastic-damage constitutive models were identified. A homogenization procedure was then employed to transfer these constituent responses to the macroscale, enabling prediction of effective composite properties and failure behavior under coupled thermo-mechanical loading. Model predictions were validated against experimental observations, including fracture morphology and stress–strain responses, demonstrating that the framework can capture temperature- and rate-dependent transitions in deformation and damage mechanisms.

Holzapfel and Gasser [147] presented a finite-strain anisotropic viscoelastic constitutive framework tailored for fiber-reinforced composites, with explicit attention to finite element implementation. The composite is modeled as a nearly incompressible matrix reinforced by two fiber families, so that the overall stress response depends on two preferred directions. For robust FE deployment, the authors derived closed-form expressions for the consistent fourth-order tangent moduli in full generality, facilitating efficient Newton-type solutions and stable large-scale simulations. They also addressed the typical incompressibility-related numerical issue by employing a mixed formulation with appropriate interpolation choices to mitigate volumetric locking. The method was demonstrated in 2D/3D FE examples, showing that the proposed computational framework can reproduce anisotropic, time-dependent responses at finite strains.

Ryou et al. [148] developed a phenomenological constitutive model tailored for woven composites and implemented it for nonlinear finite element analysis to capture three experimentally critical features: tension–compression asymmetry, anisotropic nonlinear evolution, and strain-rate dependence. Specifically, their formulation is built within an elasto–viscoplastic framework using a modified Drucker–Prager yield criterion, coupled with an anisotropic nonlinear hardening law to represent the evolving directional response under different loading modes. Material parameters were identified through a dedicated characterization procedure based on uniaxial tension and compression tests at multiple strain rates, enabling calibration of both asymmetry and rate effects. The resulting constitutive equation was then incorporated into a finite element code and validated by comparing three-point bending simulations with experimental results, demonstrating its suitability for structural-level predictions of woven composite response beyond linear elasticity.

### 3.7. Discussion

While the computational approaches reviewed in this section collectively provide a comprehensive toolkit for modeling polymer composites across multiple length scales, it is equally important to critically examine the assumptions, limitations, and damage mechanisms inherent to each modeling level. Such an assessment is essential for understanding the predictive scope of individual methods, identifying sources of uncertainty, and guiding the rational selection and integration of modeling strategies in multiscale frameworks. In this context, the following discussion synthesizes the strengths and shortcomings of atomistic, microscale, mesoscale, and continuum approaches with particular emphasis on their ability to capture damage initiation and failure processes.

Despite the complementary strengths of these computational approaches, each modeling scale is inherently associated with assumptions and limitations that constrain its predictive capability, particularly with respect to damage and failure. Constituent-scale atomistic and micromechanical models provide detailed insight into interfacial interactions and local deformation mechanisms, but are limited by accessible length and time scales and by the transferability of force fields or constituent properties to complex composite architectures. Microscale RVE-based and homogenization approaches rely on assumptions regarding representativeness, statistical dispersion, and idealized interphase behavior, which can influence predictions of stiffness, strength, and damage initiation, especially in heterogeneous or highly anisotropic systems. Mesoscale laminate and ply-level models effectively capture stacking sequence effects and interply interactions, yet typically require phenomenological damage criteria and may not explicitly resolve microcrack nucleation or fiber–matrix debonding. At the macroscale, continuum models enable efficient structural-level analysis but depend heavily on homogenized constitutive descriptions and empirical damage laws, limiting their ability to directly incorporate nanoscale-driven failure mechanisms. Recognizing these assumptions and limitations is essential for the informed selection and integration of modeling approaches and underscores the importance of multiscale frameworks that explicitly link damage processes across length scales.

Multiphysics coupling plays an important role in the modeling of polymer composites, particularly when mechanical behavior is strongly influenced by thermal, electrical, or environmental fields. Thermo-mechanical coupling is commonly employed to capture temperature-dependent stiffness [149], viscoelasticity [150], residual stresses [151] induced during curing or processing, and thermally driven damage evolution [146]. In fiber-reinforced polymer composites, such coupling is often implemented within continuum-based finite element frameworks using RVEs, homogenized constitutive laws, or $FE^{2}$-type multiscale approaches [151,152,153]. Electro-mechanical coupling is also relevant in functional composites containing conductive fibers or piezoelectric constituents, where mechanical deformation interacts with electrical fields [154,155]. In these systems, multiphysics models typically rely on well-defined constitutive relations and scale separation assumptions, enabling efficient simulation of coupled responses at the structural level while maintaining computational tractability.

Polymer composites and polymer nanocomposites share many common computational modeling frameworks; however, the underlying physical mechanisms and dominant length scales motivating these approaches differ in important ways. Conventional polymer composites are typically governed by microscale to mesoscale interactions associated with fiber or particulate reinforcements, for which continuum-based descriptions, representative volume elements, and homogenization techniques are often sufficient to capture effective mechanical behavior, interfacial load transfer, and damage evolution. In contrast, polymer nanocomposites introduce additional nanoscale phenomena arising from the high specific surface area of nanofillers, strong polymer–nanofiller interactions, confinement-induced modifications of polymer chain dynamics, and dispersion or agglomeration effects. These features frequently require molecular-level descriptions or multiscale coupling strategies that bridge atomistic, mesoscopic, and continuum models. Although similar numerical techniques may be applied across both material classes, the modeling assumptions, inputs, and scale-bridging requirements differ, motivating the distinct considerations discussed in the next chapter.

### 3.8. Summary

This section reviews the principal computational approaches used to model polymer composites across multiple length scales, spanning constituent, micro-, meso-, and macroscales, highlighting how different modeling strategies capture complementary aspects of composite behavior. At the constituent scale, molecular-level and micromechanical descriptions provide mechanistic insight into phase properties, interfacial interactions, and local deformation mechanisms that govern load transfer, damage initiation, and failure process. At the microscale, RVE-based and homogenization approaches enable explicit representation of reinforcement architecture, spatial dispersion, and orientation distributions, thereby establishing quantitative links between microstructural features and effective mechanical responses. Mesoscale models effective capture laminate stacking sequences, interply interactions, and coupled in-plane and out-of-plane responses, while macroscale continuum models facilitate structural-level analyses of composite components and assemblies at scales directly relevant to engineering design and performance evaluation.

Finally, the individual approaches introduced in this section can be systematically integrated within multiscale modeling frameworks to form coherent predictive pipelines. Representative collective efforts in this area have been summarized in several publications and also in a dedicated book chapter [156,157,158,159,160,161,162,163,164,165,166,167,168,169]. By enabling systematic information transfer across scales, multiscale models link constituent-level mechanisms, microstructural morphology, and macroscopic constitutive behavior within a unified framework. Such integration is essential for moving beyond phenomenological descriptions toward truly predictive modeling and rational, mechanics-informed design of polymer composite systems.

## 4. Computational Modeling of Polymer Nanocomposites

Polymer nanocomposites differ fundamentally from conventional polymer composites in that their reinforcing phases are characterized by at least one dimension in the nanometer range, such as nanoparticles, nanofibers, nanotubes, or nanosheets. Nanoscale reinforcements introduce new physical phenomena, including large interfacial areas, strong surface interactions, confinement effects, and size-dependent mechanical behavior, that are absent or negligible in conventional polymer composites. These features often lead to nonclassical reinforcement mechanisms, such as interphase-dominated load transfer, altered polymer chain mobility, and nonlinear or rate-dependent responses that cannot be adequately described using traditional micromechanics-based composite theories. As a result, computational modeling of polymer nanocomposites requires approaches that extend beyond classical composite theory to capture nanoscale physics. At the atomistic and molecular scales, MD and related techniques are widely employed to investigate polymer–nanofiller interfacial bonding, interphase formation, dispersion states, and the influence of surface chemistry on local mechanical and thermomechanical behavior. At the mesoscale, CG and RVE-based models are commonly used to capture filler morphology, spatial distribution, percolation behavior, and interphase overlap effects, providing an intermediate description that bridges nanoscale interactions and effective composite response. At the continuum scale, enhanced constitutive models and homogenization frameworks incorporate nanoscale information through effective material parameters, internal length scales, or interphase-informed constitutive laws to predict macroscopic stiffness, strength, and failure behavior. This section provides a comprehensive review of the principal computational modeling methodologies developed for polymer nanocomposites.

### 4.1. Atomistic MD Modeling

Atomistic MD modeling provides a physics-based framework for resolving the molecular-scale structure and dynamics of polymer nanocomposites, particularly the polymer-reinforcement interactions. In contrast to continuum and mesoscale approaches, MD modeling explicitly represents individual atoms and their interactions through interatomic potentials [170], enabling direct simulation of polymer chain conformations [171], segmental dynamics [172], interfacial interactions [173], and thermomechanical response [174] at nanometer and nanosecond scales. This capability makes MD particularly valuable for understanding the fundamental mechanisms regarding the spatial arrangement of reinforcement, polymer–reinforcement interactions, and the origin of effective nanocomposite properties.

In atomistic MD simulations of polymer nanocomposites, the polymer matrix is typically modeled using all-atom force fields, most commonly from the OPLS [175], CHARMM [176], and AMBER [177] families, as well as polymer-oriented condensed-phase force fields such as COMPASS/COMPASS II [178,179]. All-atom models capture detailed chemical specificity, including bond stretching, angle bending, torsional rotations, and nonbonded interactions, and are therefore well suited for studying interfacial chemistry, hydrogen bonding, and local chain mobility near reinforcement surfaces. Reinforcing phases such as nanoparticles, nanotubes, graphene sheets, or short fibers are explicitly included as atomistic entities within the simulation domain. Their interactions with the polymer matrix are governed by van der Waals forces [180], electrostatic interactions [181], or specific chemical bonding [182], depending on surface functionalization and material chemistry. Atomistic MD has been widely used to investigate how nano-reinforcement size, shape, surface configuration, and volume fraction influence the performance of polymer nanocomposites. A major strength of atomistic MD lies in its ability to probe interfacial phenomena, which are often inaccessible to continuum descriptions. MD studies have elucidated interphase formation and polymer immobilization near nano-reinforcement surfaces, load transfer mechanisms at the polymer–nanon-reinforcement interface, and the molecular origins of enhanced stiffness, strength, or toughness in nanocomposites. These insights provide essential guidance for the rational design and optimization of polymer nanocomposites.

Al-Ostaz et al. [183] presented an early molecular-simulation framework for evaluating the elastic and engineering properties of carbon nanotube (CNT)–polymer nanocomposites by conducting MD simulations on the individual constituents as well as on the assembled single-wall carbon nanotube (SWCNT)–polyethylene nanocomposite. Their computational strategy emphasized constituent-resolved property extraction, i.e., estimating stiffness/engineering constants for the matrix and nanotube phases separately and then for composite systems containing aligned and randomly distributed SWCNTs, enabling direct comparison of reinforcement arrangement effects at the molecular level. To probe reinforcement morphology effects beyond a single nanotube, they also simulated SWCNT bundles using the same MD-based property evaluation approach. A notable modeling detail is that the authors maintained the local density of the polymer in the vicinity of the CNT to match experimentally observed densities for similar nanocomposites, reflecting an explicit attempt to ensure realistic interfacial packing when computing effective properties. Overall, this work exemplifies a practical MD workflow for nanocomposites.

Jia et al. [184] employed fully atomistic MD simulations to investigate interfacial structure and dynamics in a polymer nanocomposite consisting of octaaminophenyl polyhedral oligomeric silsesquioxane (OAPS) nanoparticles dispersed in a poly(2-vinylpyridine) (P2VP) matrix. All constitutive atoms in both the polymer and the functionalized nanoparticle were explicitly represented using a classical force field, enabling direct resolution of polymer–particle interactions at the molecular scale. The authors systematically controlled hydrogen-bonding interactions to isolate their influence on chain packing and mobility near the nanoparticle surface. Analysis of radial density profiles and segmental dynamics revealed the formation of a ~1 nm interfacial region with altered polymer structure and dynamics relative to the bulk. The study demonstrates how atomistic MD can be used to quantify interphase thickness, polymer adsorption, and nanoparticle–polymer coupling in attractive nanocomposite systems, providing mechanistic insight into nanoscale reinforcement and transport behavior.

Lin et al. [185] investigated graphene-reinforced PMMA nanocomposites using MD simulations to quantify how graphene volume fraction and temperature affect effective elastic properties. In their computational setup, graphene nanoplatelets were assumed to be fully exfoliated and planar-oriented within the PMMA matrix, and the composite response was obtained by applying deformation simulations to extract the Young’s modulus and shear modulus over a temperature range of 300 to 500 K. To support interpretation and ensure consistent nanosheet property inputs, they also performed separate MD simulations of a single-layer graphene sheet under uniaxial tension, in-plane pure shear, and uniform transverse loading, and used these results to uniquely determine the graphene’s effective thickness and corresponding elastic constants used in the nanocomposite analysis.

MD simulations are also employed to extract effective nanocomposite material properties such as elastic moduli, thermal expansion coefficients, diffusion coefficients, and glass transition temperatures. These properties can be obtained by applying controlled deformations or thermal protocols and analyzing the resulting stress–strain or structural responses. Griebel et al. [186] conducted one of the early MD studies to quantify the elastic moduli of polymer–CNT nanocomposites by explicitly modeling a SWCNT embedded within an amorphous polymer matrix. In this framework, the composite system was constructed at the atomistic level, with the SWCNT represented by a cylindrical arrangement of carbon atoms and the surrounding polymer chains equilibrated around the reinforcement to mimic a realistic interfacial environment. Classical interatomic potentials were employed to describe both carbon–carbon interactions within the nanotube and polymer–polymer and polymer–nanotube interactions; these potentials provided the forces required to evolve atomic trajectories via Newton’s equations of motion in standard MD integration. The authors applied mechanical deformations to the equilibrated atomistic cell and monitored the resulting stress response, enabling direct computation of directional elastic moduli of the nanocomposite via stress–strain relationships under controlled strain increments. Through averaging of virial stresses and periodic boundary conditions, the effective stiffness of the composite was extracted as a function of nanotube orientation and volume fraction. This study demonstrates the use of MD as a bottom-up computational tool for capturing nanoscale reinforcement effects and interfacial mechanics at the molecular level.

In addition to equilibrium MD approaches, non-equilibrium molecular dynamics (NEMD) simulations are widely employed to investigate the rheological behavior of polymer melts and polymer-based nanocomposites. NEMD methods impose controlled deformation or flow fields, such as simple shear or uniaxial extensional flow, allowing direct evaluation of rheological quantities under non-equilibrium conditions [187]. Under shear flow, NEMD enables direct computation of shear-rate-dependent viscosity and viscometric functions while simultaneously resolving microstructural changes such as chain stretching, alignment, and entanglement disruption that underlie shear thinning in polymer melts [188]. In practice, shear NEMD is commonly implemented using Lees–Edwards sliding periodic boundary conditions together with the SLLOD equations of motion and thermostats, providing a homogeneous shear field compatible with periodic simulation cells [189]. For extensional and elongational flows, NEMD is particularly valuable because extensional viscosity and strain-hardening/softening are central to polymer processing and are often more sensitive to molecular architecture than shear viscosity. Long-time extensional simulations typically require specialized boundary conditions to prevent unbounded cell distortion while maintaining homogeneous deformation [190,191]. These methods allow the extraction of extensional viscosity and strain-rate-dependent response, and they connect macroscopic rheology to molecular-level mechanisms such as coil–stretch transitions and orientation dynamics [192]. For polymer nanocomposites, NEMD has become a practical route to quantify how nanoparticle loading, particle size, and polymer–filler interactions alter melt rheology, while also exposing the underlying physics [193,194]. In this way, NEMD provides both rheological observables relevant to processing and mechanistic insight into how molecular and interfacial structure controls flow behavior in polymer melts and nanocomposite systems [90].

Despite these strengths, atomistic MD requires substantial computational resources, which constrain both the spatial and temporal scales that can be practically simulated [195,196]. As a result, MD is most effective when used in conjunction with mesoscale or continuum methods, providing molecular-level insights and mechanistic understanding that inform constitutive modeling and homogenization at larger scales. Overall, atomistic MD plays a central role in establishing a physical understanding of polymer nanocomposite behavior and in enabling rational, bottom-up materials design.

### 4.2. CG MD Modeling

CG MD modeling [197,198] provides an intermediate description between atomistic simulations and mesoscale approaches by grouping multiple atoms into effective interaction sites, or “beads”, and employing effective potentials to describe their interactions. This reduction in degrees of freedom significantly extends the accessible length and time scales, enabling simulations of larger polymer nanocomposite systems and longer dynamic processes compared to atomistic MD.

**Figure 3:**
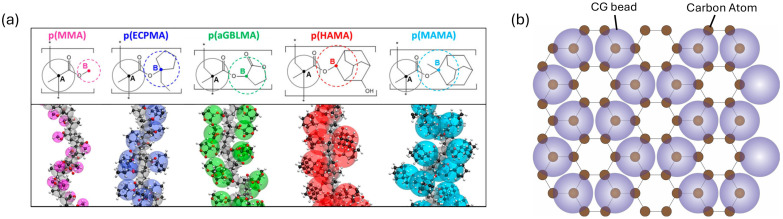
(**a**) A coarse-graining strategy for methacrylate-based polymers. Backbone CG beads (A) are shown in gray, and side-chain CG beads (B) are shown in colors. Adapted with permission from [199], Hsu, et al, 2014. (**b**) A coarse-graining strategy for graphene, with carbon atoms shown in brown and CG beads shown in purple.

In CG MD, the mapping from atoms to CG beads is performed in a manner that preserves essential structural, thermodynamic, or dynamic features of the underlying system. CG force fields are typically parameterized either by matching structural distributions, reproducing thermodynamic properties, or fitting to forces or potentials of mean force obtained from atomistic simulations. For the coarse-graining of methacrylate-based polymers, Hsu et al. [199] proposed a systematic bottom-up coarse-graining strategy in which each polymer monomer is mapped to two CG beads representing the backbone and side group, as shown in Figure 3a, thereby preserving essential molecular architecture while reducing degrees of freedom. Bonded interactions are parameterized by Boltzmann inversion of atomistic distributions, and nonbonded interactions are fitted to reproduce thermodynamic targets such as density and glass-transition temperature. The resulting model reproduces key structural and thermomechanical properties while enabling simulations at substantially larger length and time scales than atomistic MD, making it well-suited for mesoscale modeling of polymer-based systems.

On the other hand, for graphene, a highly effective nano-reinforcement, Ruiz et al. [200] developed a CG MD model specifically for multi-layer graphene based on a strain-energy–preserving coarse-graining strategy, with the objective of enabling efficient simulation of large-scale deformation and fracture processes while retaining the essential mechanical characteristics of the graphene lattice. In their approach, groups of carbon atoms in the hexagonal graphene lattice are mapped onto CG beads (4:1 mapping, as shown in Figure 3b), preserving lattice symmetry and allowing compatibility with other CG models used for polymers and biomolecules.

**Figure 4:**
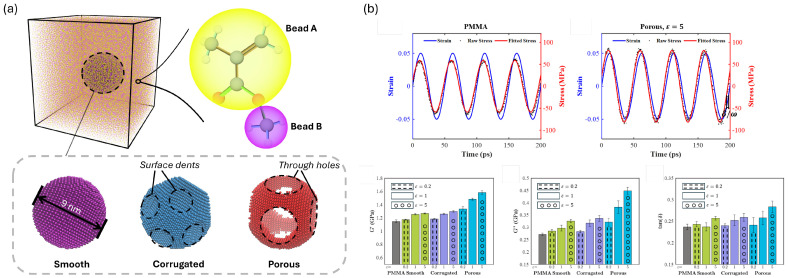
(**a**) CG models of nanoparticle-PMMA nanocomposites. (**b**) Oscillatory shear test results showing the nanoparticle-PMMA nanocomposites overcoming the conventional stiffness-damping tradeoff. Adapted from [23], Yang, et al., 2025.

In polymer nanocomposites, CG MD has been widely applied to investigate phenomena that require large system sizes or long simulation times, which are not feasible with atomistic MD yet. By explicitly representing nano-reinforcements, such as nanoparticles, nanofibers, or nanosheets, as CG objects embedded within a polymer matrix, CG MD enables investigation of how nano-reinforcement size, shape, surface condition, and loading fraction influence the mechanical behavior of nanocomposites. Yang and Meng [23] employed CG MD to investigate how nanoparticle surface morphology affects the mechanical and viscoelastic response of PMMA-based nanocomposites. Smooth, corrugated, and porous nanoparticles (Figure 4a) were explicitly modeled, and polymer–particle interfacial strength was tuned through Lennard–Jones interaction parameters. Tensile, shear, and oscillatory shear simulations were used to extract elastic and viscoelastic properties. The study showed that porous nanoparticles, which allow polymer penetration and confinement, can simultaneously enhance stiffness and damping, providing a computational demonstration of how nanoscale structural design can overcome the conventional stiffness–damping tradeoff, as illustrated in Figure 4b.

Wang et al. [201] developed an atomistically informed CG MD multiscale framework to study the temperature-dependent thermomechanical behavior of graphene-reinforced conjugated polymer nanocomposites, focusing on poly(3-alkylthiophene) (P3AT) matrices with varying side-chain lengths. Their CG strategy maps clusters of atoms to super-beads, and the authors parameterize energy-renormalization factors for P3AT CG models to achieve temperature-transferable polymer representations across different chemistries. Graphene is represented as a mechanically consistent CG sheet, and polymer–graphene nonbonded interactions are described using Lennard–Jones cross-interactions with standard combining rules; graphene sheets are randomly inserted into the polymer matrix at prescribed loadings under periodic boundary conditions to emulate bulk behavior. Using this CG MD model, the authors performed uniaxial tensile deformations to extract elastic moduli from the small-strain regime and analyzed how modulus varies with temperature, graphene content, and side-chain length; the modulus trends were additionally compared to a modified Halpin–Tsai micromechanical model. To connect macroscopic response with molecular mechanisms, they quantified graphene dispersion and characterized dynamical heterogeneity using a local molecular stiffness metric derived from the inverse Debye–Waller factor. Overall, this work exemplifies how CG MD enables efficient prediction and mechanistic interpretation of the thermomechanical behavior of graphene–polymer nanocomposites at length and time scales beyond those accessible to typical atomistic MD.

Duan et al. [202] developed and assessed a multiscale CG modeling strategy for CNT-epoxy nanocomposites with a specific focus on how to construct a physically consistent CNT–epoxy interface in CG MD. Their CG framework separates force fields into three components: (i) CNT reinforcement interactions, (ii) crosslinked epoxy matrix interactions, and (iii) CNT–epoxy interfacial interactions. The CNT CG model follows an existing mechanically calibrated CNT potential, while the epoxy CG potentials are taken from their prior work using iterative Boltzmann inversion combined with an ML-assisted parameterization. A key methodological contribution is their demonstration that accurate CG nanocomposite predictions require two interface-level equivalence conditions relative to atomistic models: preserving interfacial cohesive energy and reproducing interfacial load-transfer behavior.

CG MD is also well-suited for exploring the dynamic properties of polymer nanocomposites, which are typically difficult to access with atomistic MD due to time-scale limitations. Using CG MD, Yang et al. [19] investigated high–strain-rate piston impact in layered PMMA-graphene nanocomposite films to clarify how shock waves couple with nanoscale interfaces and govern energy dissipation. Their simulations resolve the spatiotemporal evolution of stress and local density fields, explicitly capturing the formation and interaction of a shock front, reflected wave, and release wave, and demonstrating that the layered system still follows a largely linear Hugoniot $U_{s}$–$U_{p}$ relationship commonly reported for bulk polymers.

A key mechanism that contributes to enhanced energy dissipation is the emergence of PMMA crazing once a critical piston velocity is reached, which initiates near the meeting region of the dominant reflected and release waves, markedly increasing the fraction of input energy dissipated through irreversible deformation. This dissipation ratio peaks around the crazing-onset threshold and then diminishes at higher impact velocities. The study further shows that sufficient PMMA–graphene interfacial strength is required to favor crazing over premature interfacial separation, and that layer thickness modulates both the input energy and the dissipated energy, collectively highlighting interface strength and architectural length scales as tunable levers for optimizing impact energy dissipation in layered polymer nanocomposites.

Beyond shock and impact phenomena, CG MD can also be used to investigate the viscoelastic properties of polymer nanocomposites and how they are governed by nanoscale structural features. Wang and Meng [203] employed previously developed CG models of PMMA and multilayer graphene sheets (MLGSs), together with MD simulations, to systematically quantify how graphene wrinkling and layer number regulate the elastic and viscoelastic response of graphene–polymer nanocomposites. Across a matrix of configurations spanning different graphene layer counts and wrinkle “waviness,” they reported that nanocomposite stiffness increases as graphene becomes less wavy and as the number of graphene layers increases, indicating that geometric imperfection can materially reduce the reinforcement efficiency of MLGSs. Beyond quasi-static elasticity, they observed a pronounced, sudden stress drop during out-of-plane shear for specific wrinkled configurations, and complementary small-amplitude oscillatory shear (SAOS) simulations showed unusually large loss tangents for those same cases, consistent with enhanced energy dissipation. Mechanistically, the authors attributed these dissipative signatures to the activation of interlayer sliding within wrinkled MLGSs; steered molecular dynamics measurements further supported that wrinkled sheets exhibit lower interlayer shear strength than flat counterparts. Overall, these results suggest that “wrinkle engineering” offers a practical way to control both stiffness and energy dissipation in graphene-reinforced polymer nanocomposites by modulating interlayer sliding.

Despite its clear computational advantages, CG MD models of polymer nanocomposites necessarily average out atomistic degrees of freedom, which reduces chemical specificity and can obscure interaction motifs that depend on directional or many-body physics (e.g., hydrogen bonding [204,205] or specific chemical bonding [206,207]) unless such effects are deliberately embedded in the CG potential form and parameterization [208,209,210]. As a result, CG MD force fields are commonly parameterized against higher-resolution atomistic simulations using bottom-up schemes, and they are typically validated by verifying that targeted structural/thermodynamic, or mechanical observables are accurately reproduced [208,211].

To provide an overview of the diverse MD and CG MD efforts discussed above, Table 1 summarizes representative computational studies of polymer nanocomposites reported in the literature. The table categorizes each study by material system, nano-reinforcement type, modeling method, force field or interaction potential, simulation software, and primary scientific focus. Together, these studies underscore the breadth of MD and CG MD approaches and their utility for systematically elucidating nanoscale reinforcement mechanisms in polymer nanocomposites.

### 4.3. Continuum, Mesoscale, and Scale-Bridging Modeling

Polymer nanocomposites pose distinct modeling challenges because their macroscopic mechanical and functional properties are governed by nanoscale features, including high interfacial area, interphase formation, polymer chain confinement, and nanofiller dispersion or aggregation. These nanoscale mechanisms cannot be adequately captured by classical micromechanics developed for conventional fiber-reinforced composites. As a result, modeling approaches for polymer nanocomposites have progressively evolved toward modeling frameworks that combine continuum descriptions, mesoscale representations of morphology, and explicit scale-bridging strategies to incorporate nanoscale physics into macroscopic predictions. Recent studies and reviews have emphasized that accurate prediction of polymer nanocomposites’ physical or mechanical behavior requires consistent information transfer across scales, as well as careful treatment of interphase properties and filler morphology within both continuum and mesoscale models.

#### 4.3.1. Continuum-Scale Modeling of Polymer Nanocomposites

At the continuum scale, polymer nanocomposites are typically represented through effective medium descriptions, in which the nanofillers and their surrounding interphase are incorporated into constitutive parameters used in structural analysis. Classical homogenization frameworks, such as the rule of mixtures [214], Halpin–Tsai-type relations [215], Mori–Tanaka [24], self-consistent schemes [216], and Eshelby-based inclusion models [217], are frequently adopted as baseline models, then extended to account for nanofiller geometry, imperfect interfaces, filler orientation distributions, and interphase effects. A central challenge is that polymer nanocomposite reinforcement is often dominated by interfacial physics and morphology, which are not directly captured by purely phenomenological effective property fits.

A widely used strategy is to incorporate an explicit interphase, which is a region around the nanoparticle with properties distinct from the bulk polymer, either via core–shell inclusion models where nanoparticle and interphase are treated as an effective inclusion, or interphase-augmented micromechanics that introduces interphase thickness and modulus as additional parameters. Recent work has specifically addressed the coupling between interphase size and stiffness and its consequences for predicted effective modulus, providing a route to embed interphase effects into micromechanical elastic predictions [218]. In parallel, multiscale constitutive modeling efforts have sought to infer continuum constitutive parameters from molecular simulations, enabling the continuum scale to reflect chemistry- and interface-dependent behavior rather than relying solely on calibration to macroscopic tests. Early and influential studies in this direction include molecular-informed constitutive modeling pipelines for polymer nanocomposites, where atomistic simulations are used to parametrize continuum descriptions and explore structure–property trends [219].

Continuum modeling approaches offer significant computational efficiency and natural compatibility with structural finite-element analyses, but their applicability depends strongly on assumptions concerning dispersion state, interphase representation, and interfacial mechanics. When nanoparticle networks, agglomerates, or anisotropic filler alignment dominate macroscopic response, a single effective-modulus formulation is generally insufficient unless supplemented by explicit morphological descriptors or multiscale-informed constitutive parameters.

#### 4.3.2. Mesoscale Modeling of Polymer Nanocomposites

Mesoscale approaches aim to resolve representative microstructures and explicitly represent morphology, enabling mechanistic prediction of stress/strain localization, load transfer pathways, and damage initiation driven by nanoscale dispersion states. In practice, mesoscale polymer nanocomposite modeling commonly employs RVEs containing explicit fillers with prescribed orientation distributions and spatial statistics. The polymer matrix is modeled as elastic, viscoelastic, or elasto-plastic, and the nanoparticle–matrix interface is represented through cohesive laws, interphase layers, or contact/friction models.

A recurring methodological issue is the choice and validation of RVE size and the number of random realizations needed to obtain converged effective responses for heterogeneous polymer nanocomposites. Statistical investigations have shown that the smallest RVE size and the required number of realizations depend strongly on the targeted response and desired precision, underscoring that mesoscale predictions can be biased if insufficient sampling is used [220]. Mesoscale models are also well suited for studying non-mechanical effective properties such as permeability/diffusion in barrier nanocomposites [221].

Interphase effects can be included at the mesoscale by explicitly meshing a shell region with distinct properties around each filler, allowing one to explore interphase thickness, stiffness gradients, and their coupling to filler size and volume fraction. Recent FE frameworks that integrate interphase modulus–size interdependency into micromechanical prediction highlight the importance of considering interphase parameters as scale-dependent rather than fixed constants [218]. Despite their enhanced fidelity, mesoscale methods remain computationally intensive as filler content, aspect ratio, and spatial resolution increase, and they often require careful calibration of interphase and interface parameters to avoid purely empirical tuning.

#### 4.3.3. Scale-Bridging in Modeling of Polymer Nanocomposites

Scale-bridging approaches address the core limitation of continuum and mesoscale models: their reliance on interphase/interface properties and morphology descriptors that are difficult to determine experimentally and can be highly system-specific. In polymer nanocomposites, bridging is commonly performed through hierarchical “bottom-up” workflows in which MD or CG MD provide inputs to higher-scale models, including: (i) effective filler and interphase elastic constants, (ii) interface traction–separation laws or adhesion energies, (iii) temperature-dependent viscoelastic response, and (iv) parameters for continuum constitutive models. Reviews of MD simulations of polymer-matrix nanocomposites highlight both the progress and the persistent challenges in capturing dispersion, interphase formation, and long-time dynamics, motivating the use of CG models and multiscale coupling when atomistic MD is prohibitive [90].

A representative example of an explicit multiscale interphase characterization is the modeling of crosslinked epoxy nanocomposites, where nanoscale interfacial behavior and interphase properties are characterized and subsequently integrated into micromechanical/continuum predictions. More broadly, multiscale constitutive modeling studies have demonstrated workflows that connect molecular structure and nanoscale interactions to bulk mechanical properties through parameter passing and systematic sensitivity studies [219].

There are three commonly used classes of bridging strategies in polymer nanocomposites modeling:

1. Sequential parameter passing: MD/CGMD → interphase/interface properties → mesoscale RVE → continuum FE. This is the most widely used approach but requires careful uncertainty propagation because errors in interfacial parameters can amplify at higher scales [222].
2. Concurrent coupling: local regions treated with molecular resolution embedded within a continuum domain. While conceptually appealing for capturing localized interfacial processes, concurrent schemes are often difficult to deploy for large systems due to computational cost and coupling complexity [210].
3. Reduced order and surrogate bridging: molecular and mesoscale simulations used to train surrogates that rapidly predict effective properties as functions of morphology descriptors, such as volume fraction, aspect ratio, and dispersion metrics. This approach is increasingly attractive for design and optimization but demands careful validation and physically consistent feature selection [195].

Overall, continuum, mesoscale, and bridging approaches provide complementary modeling capabilities for polymer nanocomposites. Continuum models enable rapid structural-scale evaluation but require robust parameterization; mesoscale RVE methods provide explicit morphology-driven mechanistic insight but are computationally heavier and sensitive to sampling; and scale-bridging frameworks offer a principled route to connect nanoscale physics to macroscopic behavior, though they introduce challenges in transferability, uncertainty quantification, and validation.

### 4.4. Multiscale Modeling of Polymer Nanocomposites

Polymer nanocomposites exhibit structure–composition-property relationships that span multiple length and time scales, from molecular-level interfacial interactions and chain dynamics to mesoscale nano-reinforcement dispersion and macroscopic mechanical response. While atomistic and CG simulations provide critical insight into nanoscale mechanisms, they are inherently limited in their ability to directly simulate structural-scale behavior. Multiscale modeling frameworks, therefore, play a central role in bridging molecular-level physics with continuum-level constitutive behavior and enabling predictive modeling of polymer nanocomposites across scales.

In bottom-up multiscale modeling approaches, information flows sequentially from lower to higher scales. Atomistic simulations are commonly used as the foundation for CG model construction and parameterization, providing molecular-level descriptions of polymer–reinforcement interactions, interphase characteristics, reinforcement mechanical properties, interfacial slip or friction, and local failure processes [199,223,224]. CG modeling is particularly effective at capturing mesoscale structure and composition characteristics, where it can explicitly represent nano-reinforcement morphology and collective organization phenomena, such as geometry, dispersion quality, orientation distributions, and agglomeration or network formation, that are difficult to cover with fully atomistic MD because they require much larger system sizes and longer equilibration times [90,225,226]. The results obtained from CG modeling are commonly used to inform and construct continuum constitutive material models, in which the effective material response may be expressed as a function of spatial position to reflect local variations in nano-reinforcement dispersion, orientation, and morphology, rather than assuming uniform and homogeneous reinforcement distributions. This spatially resolved constitutive description enables continuum-scale simulations to account for nano-reinforcement heterogeneity and to predict their influence on macroscopic mechanical behavior.

Zeng et al. [227] presented a comprehensive multiscale perspective on computational modeling of polymer nanocomposites, emphasizing that predictive understanding requires linking phenomena across molecular, mesoscopic, and continuum scales. The review surveys the major simulation and theory toolsets used in this field, from molecular-scale methods that resolve polymer–reinforcement interactions and interphase effects, to mesoscale descriptions that capture dispersion/aggregation and morphology evolution, and onward to continuum and micromechanics frameworks for effective-property prediction and structural-scale analysis. The authors highlight how modeling has been applied to understand the thermodynamics and kinetics of reinforcement incorporation, polymer structure and dynamics near interfaces, morphology and processing behaviors, and the resulting mechanical performance, while also emphasizing the need for robust cross-scale parameter passing and validation to make multiscale workflows truly predictive.

Sun et al. [156] presented a bottom-up multiscale modeling framework for predicting failure behavior in carbon fiber–reinforced polymer composites by explicitly integrating physics and parameters across four coupled length scales. At the nanoscale, MD simulations, together with an analytical gradient description, were used to determine the mechanical properties of the fiber-matrix interphase; these interphase attributes were then embedded into microscale RVE models to predict the failure strength and failure envelopes of unidirectional fiber-reinforced polymer composites. The microscale results were subsequently leveraged to develop an elastic–plastic–damage constitutive law for fiber tows in woven composites, enabling efficient representation of mesoscale structural elements and facilitating mesoscale RVE predictions of woven-composite failure mechanisms and strength. Finally, the authors demonstrated that a homogenized macroscale model, built upon these lower-scale components, can capture the structural response of a U-shaped part under four-point bending, with reported agreement between simulations and experiments across multiple scales. Although the reinforcements considered in this study are not nanoscale in size, the multiscale modeling methodology presented is general in nature and can be applied to nanocomposite and other hierarchical material systems.

Overall, multiscale modeling provides a unifying framework that integrates atomistic, CG, and continuum descriptions into a coherent predictive pipeline. By systematically transferring information across scales, multiscale approaches enable quantitative prediction of macroscopic behavior based on nanoscale structure and composition, thereby supporting rational materials design and optimization of polymer nanocomposites for targeted mechanical and functional performance.

### 4.5. Discussion

The computational approaches reviewed in this section demonstrate the significant progress that has been made in modeling polymer nanocomposites across molecular and mesoscale regimes. At the same time, a critical examination of their underlying assumptions and limitations is essential for assessing their predictive scope and guiding future methodological developments. In polymer nanocomposites, where macroscopic behavior is highly sensitive to nanoscale morphology, interfacial chemistry, and dynamic processes, modeling accuracy depends not only on resolution but also on how information is transferred and interpreted across scales.

Atomistic molecular dynamics simulations provide unparalleled insight into polymer–nanoreinforcement interactions, interfacial adhesion, chain confinement, and local deformation mechanisms. However, their predictive capability is constrained by accessible length and time scales, as well as by the transferability of force fields across different polymer chemistries, filler surfaces, and environmental conditions. Damage and failure processes captured at the atomistic scale are often highly localized and system-specific, making direct extrapolation to macroscopic failure behavior nontrivial without systematic coarse-graining or homogenization strategies.

CG molecular dynamics extends modeling capability to larger system sizes and longer times, enabling explicit investigation of nanofiller dispersion, aggregation, orientation, and collective dynamics. Nevertheless, CG models introduce additional assumptions through reduced resolution and parameterization, which can obscure or average out critical chemical details governing interfacial strength and failure. The extent to which CG interaction potentials preserve damage-relevant mechanisms, such as progressive interfacial degradation or strain rate-dependent behavior, depends strongly on the chosen coarse-graining strategy and calibration procedure. As a result, careful validation against atomistic simulations or experimental testing remains essential.

A central challenge in multiscale modeling of polymer nanocomposites lies in the transfer of damage mechanisms across scales. While molecular simulations can identify nanoscale precursors to damage, higher-scale models often rely on phenomenological constitutive laws or empirical failure criteria that do not explicitly encode these mechanisms. This disconnect can limit predictive accuracy, particularly for properties governed by interfacial failure, time-dependent viscoelasticity, or nonlinear damage accumulation. Bridging frameworks that systematically link molecular insights to mesoscale morphology descriptors and continuum damage models represent a promising direction, but they remain sensitive to assumptions regarding representativeness, parameter uniqueness, and uncertainty propagation.

In polymer nanocomposites, multiphysics interactions are often governed by nanoscale phenomena that require more detailed descriptions and tighter coupling across length scales. The high specific surface area of nanofillers amplifies interfacial effects, leading to strong coupling between mechanical behavior and thermal transport, electrical conductivity, or electromagnetic response [61,228,229]. Thermo-mechanical coupling in nanocomposites must often account for confinement-induced changes in polymer chain mobility, altered glass transition behavior, and non-uniform temperature or stress fields near interfaces. Similarly, electro-mechanical coupling in nanocomposites with conductive or dielectric nanofillers is strongly influenced by filler dispersion, percolation networks, and interfacial polarization, which cannot be adequately captured using purely homogenized continuum models [230,231,232]. As a result, multiphysics modeling of polymer nanocomposites frequently employs multiscale strategies that integrate molecular dynamics, coarse-grained simulations, or micromechanical models with continuum formulations to accurately represent coupled field effects and emergent nanoscale mechanisms.

### 4.6. Summary

This section reviews the principal computational methodologies for polymer nanocomposites, with emphasis on atomistic MD, CG MD, and their integration within multiscale modeling frameworks. Atomistic MD simulations provide detailed insight into polymer–nano-reinforcement interactions, chain dynamics, and local deformation and failure mechanisms, enabling quantitative characterization of interfacial strength, confinement effects, and nanoscale structure–property relationships. These molecular-level descriptions establish a mechanistic foundation for understanding how nano-reinforcements alter polymer behavior and how interfacial phenomena ultimately govern macroscopic performance. CG MD extends this capability to larger system sizes and longer time scales, enabling systematic investigation of nano-reinforcement dispersion, orientation, aggregation, and collective dynamic behavior, as well as phenomena such as viscoelastic relaxation and high–strain-rate response that are inaccessible to atomistic simulations. By explicitly representing nano-reinforcements and their interactions with the polymer matrix at reduced resolution, CG MD captures essential mesoscale morphology and dynamics while retaining mechanistic connections to the underlying molecular mechanisms.

The section further discusses how atomistic and CG models are integrated within multiscale frameworks to enable predictive modeling across length and time scales. In these approaches, MD simulations inform CG models and continuum constitutive laws through systematic transfer of interfacial properties, phase behavior, and damage mechanisms. Such cross-scale integration allows modeling efforts to move beyond phenomenological descriptions toward physically grounded, predictive frameworks. By connecting mechanisms across molecular, microstructural, and continuum scales, these models can describe how interfaces, heterogeneity, anisotropy, and time-dependent effects work together to determine the behavior of polymer nanocomposites.

Overall, computational modeling of polymer nanocomposites has evolved into a powerful framework for gaining mechanistic insight, enabling quantitative prediction, and guiding the rational design of nanocomposite materials. Continued advances in model fidelity, parameter transferability, validation strategies, and integration with data-driven methods are expected to further enhance predictive capability and broaden the real-world impact of these approaches.

## 5. Machine Learning for Modeling Polymer Composites and Nanocomposites

Recent advances in machine learning (ML) have introduced powerful tools for modeling polymer composites and nanocomposites, particularly in regimes where traditional simulations are computationally expensive or insufficiently predictive. By learning complex, nonlinear mappings from data, ML models offer the potential to accelerate multiscale analysis, enable efficient surrogate modeling, and support inverse design of composite systems. At the same time, the successful application of ML in this domain requires careful consideration of data availability, model validation, physical consistency, and integration with established mechanics-based approaches.

### 5.1. Data Requirements and Model Development

A central challenge in applying ML to modeling polymer composites and nanocomposites lies in the availability and quality of training data. Unlike image or text-based ML applications, materials modeling datasets are often limited in size, heterogeneous in origin, and expensive to generate. Data sources typically include high-fidelity simulations (e.g., molecular dynamics, coarse-grained models, finite-element analyses), experimental measurements, or hybrid datasets combining both. For nanocomposites, generating sufficiently diverse datasets that span variations in filler type, morphology, dispersion state, interfacial properties, and loading conditions remains nontrivial.

The choice of input features is therefore critical. Common descriptors include material composition, filler volume fraction, aspect ratio, orientation distributions, interfacial parameters, and microstructural statistics derived from representative volume elements [233,234,235]. More recent approaches employ field-based inputs, such as stress, strain, or microstructure images, using convolutional neural networks (CNNs) or graph-based models to capture spatial correlations [236,237]. Regardless of the architecture, careful feature engineering or representation learning is essential to ensure that ML models capture physically meaningful dependencies rather than spurious correlations [238].

Data efficiency is another key concern. Given the limited size of many materials datasets, techniques such as transfer learning, active learning, and physics-informed regularization are increasingly used to reduce data requirements [239,240,241]. These strategies allow ML models to leverage prior knowledge from related systems or to selectively sample new data where model uncertainty is highest.

### 5.2. Model Validation and Generalization

Robust validation is essential to ensure that ML models provide reliable predictions beyond the training dataset. In polymer composite and nanocomposite modeling, validation must address not only standard statistical metrics, such as prediction error or coefficient of determination, but also physical plausibility and generalization across material systems and loading conditions.

Cross-validation and independent test sets are commonly employed [242]; however, care must be taken to avoid data leakage, particularly when multiple samples share similar microstructures or simulation parameters. For multiscale datasets, it is often more informative to validate models on unseen combinations of microstructural parameters or loading conditions rather than random data splits.

Equally important is extrapolation behavior. ML models trained on limited parameter ranges may produce unphysical predictions when applied outside the training domain. As a result, uncertainty quantification techniques, such as Bayesian neural networks, ensemble methods, or Gaussian process regression, are increasingly incorporated to assess prediction confidence and identify regions where ML predictions should be treated with caution [243,244,245].

### 5.3. Physical Consistency and Interpretability

A major limitation of purely data-driven ML models is their lack of inherent physical constraints. Without explicit enforcement, ML predictions may violate fundamental principles such as symmetry, objectivity, conservation laws, or thermodynamic consistency. This issue is particularly critical for mechanics-based applications, where unphysical behavior can compromise downstream simulations or design decisions.

To address this challenge, several strategies have been proposed. Physics-informed machine learning approaches incorporate governing equations, constitutive constraints, or invariance principles directly into the learning process, either through tailored loss functions or architecture design [246,247,248]. For example, stress–strain relationships may be constrained to satisfy material frame indifference, or energy-based formulations may be used to ensure thermodynamic consistency.

Interpretability is another important consideration. In engineering applications, ML models are most valuable when they provide insight into structure-composition–property relationships rather than acting as black boxes. Techniques such as sensitivity analysis [249], feature importance ranking [250], and latent space visualization [251] can help identify dominant microstructural parameters and reinforce mechanistic understanding. Hybrid models that combine reduced-order physics-based representations with ML components are particularly attractive in this regard [252,253].

### 5.4. Integration with Physics-Based Multiscale Models

**Figure 5:**
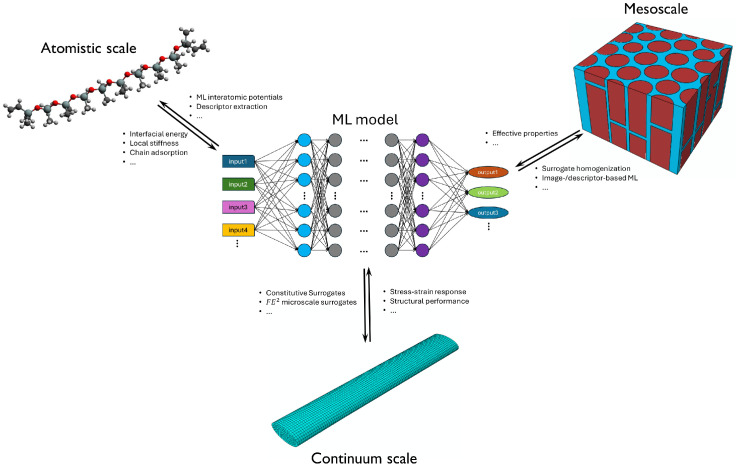
Schematic overview of how ML is integrated across atomistic, mesoscale, and continuum scales for modeling polymer composites and nanocomposites.

ML is increasingly integrated into physics-based multiscale modeling frameworks as a complementary tool that enhances efficiency, scalability, and predictive capability without replacing established mechanistic descriptions. In the context of polymer composites and nanocomposites, ML enables the acceleration of computationally intensive simulations, facilitates information transfer across length scales, and supports data-driven design within physically grounded workflows. As illustrated schematically in Figure 5, ML can be embedded at different scales of the multiscale modeling, ranging from atomistic-scale surrogates and descriptor extraction to mesoscale homogenization and continuum-level constitutive modeling.

Building on this framework, the following subsections discuss three primary modes of ML integration: surrogate modeling to approximate expensive simulations, scale bridging to translate lower-scale descriptors into higher-scale effective properties, and inverse design to identify microstructures or compositions that achieve targeted macroscopic performance under physical constraints. Together, these approaches highlight the role of ML as an enabling intermediary that complements physics-based multiscale models and expands their applicability to complex composite systems.

#### 5.4.1. Machine Learning for Surrogate Modeling Multiscale Frameworks

Rather than replacing traditional physics-based modeling approaches, ML is increasingly viewed as a complementary component within multiscale modeling frameworks, where it is used to accelerate, augment, or interpolate between established mechanistic descriptions. A particularly widespread and successful application is surrogate modeling, in which ML models are trained to approximate the input–output mappings of computationally expensive simulations across atomistic, mesoscale, or continuum scales.

At the atomistic scale, ML surrogates are often used to replace or accelerate ab initio or molecular dynamics calculations while retaining near-first-principles accuracy. For example, Behler and Parrinello [254] introduced high-dimensional neural network potentials that learn atomic energy contributions consistent with quantum-mechanical reference data, enabling simulations at length and time scales inaccessible to density functional theory alone. Subsequent developments, such as Gaussian approximation potentials (GAPs) [255] and moment tensor potentials [256], further demonstrated that ML-based interatomic models can achieve chemical accuracy while reducing computational cost by several orders of magnitude, making large-scale sampling and parametric exploration feasible. Importantly, these approaches do not discard physics; instead, they embed physical symmetries directly into the surrogate representation.

At the mesoscale, surrogate modeling has been widely adopted to approximate microstructure-resolved simulations, such as RVE-based finite element (FE) analyses. Studies by Bostanabad et al. [257] and others [258,259] demonstrated that ML models trained on homogenization datasets can rapidly predict effective elastic, plastic, or viscoelastic properties as functions of microstructural descriptors, including phase volume fraction, aspect ratio, or orientation statistics. More recent image-based approaches employ CNNs to map voxelized or image-based microstructures directly to effective responses, bypassing repeated FE solves [260,261]. These ML surrogates preserve the underlying physics implicitly learned from high-fidelity simulations while enabling large-scale parametric sweeps, uncertainty quantification, and inverse microstructure design that would otherwise be computationally prohibitive.

At the continuum scale, ML surrogates are increasingly used to emulate nonlinear constitutive responses, history-dependent behavior, or multiscale coupling terms. For example, Ghaboussi et al. [262,263] showed that neural networks can approximate complex constitutive mappings derived from micromechanics or multiscale simulations, serving as fast evaluators within finite element solvers. More recent work integrates ML surrogates into multiscale $FE^{2}$ frameworks, where the microscale problem is replaced by a trained surrogate that returns stresses, tangents, or internal variables at a fraction of the original computational cost [264,265]. This strategy preserves the hierarchical multiscale structure while enabling simulations of large structures or long time horizons.

Across all scales, surrogate modeling enables dramatic reductions in computational cost while maintaining acceptable accuracy within the trained domain. These efficiency gains are crucial for computationally intensive tasks such as design optimization, microstructure tailoring, and property-composition screening, where thousands to millions of model evaluations may be required. Consequently, ML surrogates are increasingly viewed not as black-box replacements, but as enabling technologies that allow physics-based multiscale models to be deployed in regimes that were previously infeasible.

#### 5.4.2. Machine Learning for Multiscale Bridging and Scale Coupling

ML models are increasingly used to bridge length scales by learning mappings between lower-scale descriptors and higher-scale effective properties, thereby enabling efficient information transfer across atomistic, mesoscale, and continuum descriptions. Rather than acting as standalone predictors, ML models in this context serve as translators between scales, encoding complex, high-dimensional nanoscale or microscale information into reduced representations that can be directly utilized by higher-level models. This strategy is particularly attractive in polymer composites and nanocomposites, where interfacial physics and heterogeneous microstructures play a dominant role in macroscopic response.

At the nanoscale, MD and CG simulations provide detailed descriptors of interfacial behavior, such as adhesion energy, interphase stiffness, chain adsorption statistics, and stress transfer efficiency between polymer matrices and fillers. Several studies have demonstrated that ML models can be trained on such simulation data to map these nanoscale descriptors to effective mesoscale constitutive parameters, including elastic moduli, yield properties, or viscoelastic relaxation times. For example, it has been shown that neural networks can efficiently encode atomistic deformation mechanisms and interfacial interactions into reduced constitutive descriptions, enabling rapid upscaling of nanoscale physics without repeated molecular simulations [266,267].

At the mesoscale and continuum scales, these ML-inferred constitutive parameters are passed into micromechanical models, homogenization frameworks, or FE solvers. Studies by Bostanabad et al. [257], Cecen and co-workers [268], and Mozaffar et al. [269] demonstrated that ML can link lower-scale descriptors, such as volume fraction, orientation statistics, or nanoscale-informed interphase properties, to macroscopic stress–strain responses while preserving history dependence and nonlinearity. In this multiscale setting, ML accelerates scale coupling and parameter transfer while retaining the structure of physics-based models, reinforcing its role as an enabling intermediary rather than a replacement for mechanistic understanding.

#### 5.4.3. Machine Learning-Driven Inverse Design of Materials

Inverse design represents another promising application of machine learning in multiscale materials modeling, particularly when coupled with optimization algorithms to identify microstructural configurations or material compositions that achieve targeted macroscopic performance. In contrast to forward surrogate modeling, inverse design frameworks seek to solve the ill-posed problem of determining the microstructure–composition space that produces a desired response, such as stiffness, toughness, energy dissipation, or multifunctional performance. ML models play a central role in this context by providing fast, differentiable mappings between microstructural descriptors and macroscopic properties, which can be efficiently embedded within gradient-based or evolutionary optimization loops.

Several studies have demonstrated the feasibility of ML-driven inverse design in heterogeneous materials. For example, Bostanabad et al. [257] developed data-driven frameworks that combine microstructure descriptors with surrogate models to enable inverse exploration of microstructural design spaces, allowing targeted tuning of effective properties while respecting manufacturability constraints. Similarly, Cang et al. [260] employed deep learning models to reconstruct or generate microstructures with prescribed effective responses, illustrating how learned latent representations can be navigated to achieve desired macroscopic behavior. In polymer composites and nanocomposites, such approaches have been used to identify optimal filler volume fraction, aspect ratio, orientation distribution, and spatial arrangement to balance stiffness, strength, and toughness [259,270].

More recent work has emphasized the importance of embedding inverse design within physics-based constraints to ensure physical realism and robustness. Studies by Karniadakis et al. [271] and Ferreira et al. [272] demonstrated that incorporating governing equations, constitutive consistency, or thermodynamic constraints into the learning or optimization process can significantly improve generalization and prevent nonphysical solutions. For instance, Bessa et al. [273] introduced a framework for materials design that couples ML surrogates with constrained optimization, enabling the discovery of microstructures that satisfy both performance targets and physical admissibility. Within this paradigm, ML does not replace physics-based reasoning; instead, it accelerates the search over high-dimensional design spaces while relying on mechanistic constraints to guide and regularize the solution, making inverse design a practical and reliable tool for advanced material development.

To synthesize the diverse ways in which ML is integrated into physics-based multiscale modeling frameworks, Table 2 summarizes the primary roles of ML in modeling polymer composites and nanocomposites. The table highlights how different ML applications contribute to improved efficiency, predictive capability, and design flexibility, while also outlining their key advantages and associated challenges.

### 5.5. Hybrid Data-Driven Mechanics for Fracture and Damage Evolution

Hybrid data-driven mechanics has emerged as a promising framework for modeling fracture and damage evolution in polymer composites and nanocomposites, particularly in regimes where multiscale interactions, complex microstructures, and uncertain material parameters limit the predictive capability of purely physics-based models. In these materials, damage processes often involve coupled mechanisms such as matrix cracking, interfacial debonding, filler network disruption, viscoelastic or rate-dependent deformation, and environmental or thermal effects, which are difficult to represent accurately using closed-form constitutive relations alone. Conversely, purely data-driven approaches may suffer from limited training data, a lack of physical interpretability, and poor extrapolation beyond the conditions represented in the dataset.

Hybrid approaches seek to combine the strengths of both paradigms by embedding physics-based constraints, such as energy balance, thermodynamic consistency, fracture criteria, or known constitutive relationships, within data-driven models or by using machine learning to augment specific components of established mechanics frameworks. In the context of fracture and damage, machine learning has been used to learn effective traction–separation laws, damage driving forces, or crack propagation tendencies from simulation or experimental data, while the global equilibrium, kinematics, and boundary conditions are enforced through continuum finite element or phase-field formulations [274,275,276]. This strategy allows complex, microstructure-sensitive behaviors to be captured without sacrificing physical consistency at the structural level.

For polymer nanocomposites, hybrid data-driven mechanics is particularly attractive due to the strong influence of nanoscale features such as filler dispersion, agglomeration, interphase properties, and percolation networks on damage initiation and evolution. Data-driven surrogates trained on molecular dynamics, micromechanical simulations, or high-resolution experimental data can be used to inform effective damage parameters or field-dependent constitutive responses within continuum fracture models [196]. Field-based learning approaches, including convolutional neural networks and graph-based models, have also been explored to predict damage localization or crack path evolution from stress, strain, or microstructure descriptors, enabling efficient scale bridging in multiscale simulations [277,278].

Recent developments have also emphasized uncertainty-aware and adaptive hybrid frameworks, where Bayesian inference, ensemble learning, or active learning strategies are employed to quantify prediction confidence and guide additional data acquisition in regions associated with high damage sensitivity or model uncertainty [241,279,280]. Such approaches are particularly relevant for fracture modeling, where small variations in microstructure or loading conditions can lead to markedly different failure paths. While hybrid data-driven mechanics is still an emerging area, it offers a powerful and flexible pathway for improving predictive accuracy and computational efficiency in fracture and damage modeling of polymer composites and nanocomposites, provided that careful attention is given to physical interpretability, data quality, and validation across relevant loading and environmental conditions.

### 5.6. Outlook and Challenges

Despite their promise, ML-based approaches for polymer composites and nanocomposites face several open challenges. Limited and biased datasets, difficulties in extrapolation, and the need for physically interpretable predictions remain significant hurdles. Moreover, integrating ML models seamlessly into established simulation workflows requires careful attention to numerical stability, error propagation, and compatibility with existing FE or multiscale solvers.

Future progress is likely to depend on closer integration between ML and mechanics-based modeling, with increased emphasis on physics-informed learning, uncertainty quantification, and hybrid frameworks. As these approaches mature, ML has the potential to become a valuable component of predictive multiscale modeling pipelines for polymer composites and nanocomposites, complementing traditional methods and enabling new capabilities in analysis and design.

## 6. Conclusions and Discussion

This review has synthesized the current understanding of the mechanisms governing polymer composite and nanocomposite behavior, as well as the multiscale computational approaches used to predict, interpret, and design these materials across multiple length and time scales. By first outlining the essential physical characteristics governing polymer composites and nanocomposites, the review established the physical basis underlying the complex mechanical and functional responses observed in these materials. Building on this foundation, computational approaches spanning the atomistic, CG, mesoscale, and continuum levels were examined, with emphasis on their respective capabilities, limitations, and complementary roles within integrated multiscale modeling frameworks.

At the molecular and nanometer scale, atomistic MD simulations provide critical insight into how chemistry, surface functionalization, and molecular architecture control interphase structure, load transfer, and local deformation mechanisms, while supplying quantitative parameters for higher-scale models. CG MD extends these capabilities to larger system sizes and longer time scales, enabling systematic investigations of reinforcement dispersion, orientation, agglomeration, and collective dynamic phenomena such as viscoelastic relaxation, damage initiation, and impact response. Together, atomistic and CG methods serve as essential tools for nanoscale modeling, linking molecular-level interactions to emergent mesoscale structure and behavior. These lower-scale descriptions are subsequently embedded within continuum and mesoscale frameworks to enable structural-level analysis and design. Such multiscale integration is particularly critical for polymer composites and nanocomposites, where interfacial effects, heterogeneity, anisotropy, and pronounced time-dependent behavior dominate macroscopic response and cannot be reliably captured by single-scale models.

Despite substantial progress, several challenges continue to limit the predictive capability and widespread adoption of multiscale modeling approaches. Rigorous quantitative validation against experimental data across length and time scales remains limited, especially for failure, fatigue, impact, and long-term durability. The transferability of CG and continuum parameters across different polymer chemistries, reinforcement types, processing histories, and environmental conditions also remains an open issue. In addition, the computational cost associated with high-fidelity multiscale simulations constrains their routine use in design optimization, uncertainty quantification, and large-scale parametric studies.

Looking forward, emerging opportunities lie in the integration of physics-based multiscale modeling with ML techniques to accelerate surrogate modeling, inverse design, and uncertainty-aware optimization. Equally important is the tighter coupling of processing simulations, microstructure evolution, and performance prediction to establish end-to-end, predictive digital design frameworks for polymer composites and nanocomposites. Advances in experimental characterization and data-driven validation will further strengthen the reliability and impact of these approaches.

In summary, computational modeling has become an indispensable tool for understanding, predicting, and designing polymer composites and nanocomposites. Continued progress in multiscale methodologies, ML-enabled acceleration, and cross-scale validation will further enhance their role in enabling the rational development of next-generation composites and nanocomposites with tailored, reliable, and multifunctional performance.

## Figures and Tables

**Table 1:** Summary of representative computational studies on polymer nanocomposites.

System	Naon- Reinforcement	Method	Force Field/ Potential	Software	Focus	Ref.
SWCNT–polymer nanocomposite	SWCNT	MD	COMPASS	*Materials Studio*	Elastic and engineering properties	[183]
Grafted OAPS nanoparticle nanocomposite	Grafted nanoparticles	MD	OPLS-AA	*GROMACS*	Interfacial structure and dynamics	[184]
Graphene-reinforced polymer nanocomposite	Graphene	MD	COMPASS	*LAMMPS*	Temperature-dependent mechanical properties	[185]
CNT-polymer nanocomposite	CNT	MD	REBO; LJ	In-house code	Elastic moduli	[186]
layered polymer-graphene nanocomposite film	Graphene	CG MD	MARTINI-type CG; custom LJ	*LAMMPS*	Impact response	[19]
Porous nanoparticle nanocomposite	Nanoparticles	CG MD	Custom CG; LJ	*LAMMPS*	Stiffness-damping tradeoff	[23]
Wrinkled graphene polymer nanocomposite	Wrinkled graphene	CG MD	Custom CG; LJ	*LAMMPS*	Viscoelastic properties	[203]
Fullerene $C_{60}$ polymer nanocomposite	Fullerene $C_{60}$	CG MD	IBI-derived CG potential	*N/A*	Diffusive properties	[212]
Stacked and grafted graphene/graphene oxide polymer nanocomposite	Graphene/graphene oxide	CG MD	Drieding united-atom CG potential; LJ	*LAMMPS*	Mechanical and viscoelastic properties	[213]

Note: *N/A* denotes information that is not available or not reported in the original reference.

**Table 2:** Roles of machine learning in modeling polymer composites and nanocomposites.

ML Application	Primary Function	Key Advantage	Key Challenge
Physics-informed ML	Enforce physical consistency	Prevents nonphysical predictions	Model formulation complexity
Surrogate modeling	Approximate expensive simulations	Orders-of-magnitude speed up	Limited extrapolation
Scale bridging	Map lower-scale descriptors to higher-scale properties	Efficient multiscale coupling	Loss of interpretability if unconstrained
Inverse design	Identify structure/composition for target performance	Enables optimal materials design efficiently	Optimization instability and local minima
Model validation & UQ	Assess reliability and extrapolation	Improves robustness	Added computational complexity
Data-driven feature learning	Learn representations from limited data	Reduces manual feature engineering	Risk of spurious correlations

## Data Availability

The original contributions presented in this study are included in the article. Further inquiries can be directed to the corresponding author.
